# Supplementary material for: Cardiomyocyte contractile impairment in heart failure results from reduced BAG3-mediated sarcomeric protein turnover
Source: Nat Commun. 2021 May 19;12:2942. doi: 10.1038/s41467-021-23272-z (PMC8134551; doi:10.1038/s41467-021-23272-z)
Supplement: Supplementary file 1 — Supplementary Information [file 41467_2021_23272_MOESM1_ESM.pdf]

SUPPLEMENTARY INFORMATION

Cardiomyocyte Contractile Impairment in Heart Failure Results from Reduced BAG3-mediated Sarcomeric Protein Turnover

Thomas G. Martin[1], Valerie D. Myers[2], Praveen Dubey[2], Shubham Dubey[2], Edith Perez[1], Christine S. Moravec[3], Monte S. Willis[4], Arthur M. Feldman[2], and Jonathan A. Kirk[1†]

1. Department of Cell and Molecular Physiology, Loyola University Stritch School of Medicine, Maywood, IL

2. Department of Medicine, Temple University Lewis Katz School of Medicine, Philadelphia, PA

3. Department of Medicine, Cleveland Clinic Lerner College of Medicine, Cleveland, OH

4. Department of Pathology and Laboratory Medicine, Indiana University School of Medicine, Indianapolis, IN

Supplementary Table 1. Chemical composition of the Activating, Relaxing, and Isolation solutions used for skinned myocyte functional assessment.

Activating	Final Conc. (mM)	Relaxing	Final Conc. (mM)	Isolation	Final Conc. (mM)
Ca^{2+}-EGTA	10	EGTA	10	EGTA	2
Potassium Propionate	28.1	Potassium Propionate	47.6	Potassium Hydroxide	8.9
BES	100	BES	100	Imidazole	10
$MgCl_2$	6.2	$MgCl_2$	6.5	$MgCl_2$	7.1
ATP	6.3	ATP	6.2	ATP	5.8
Creatine Phosphate	10	Creatine Phosphate	10	Potassium Chloride	108

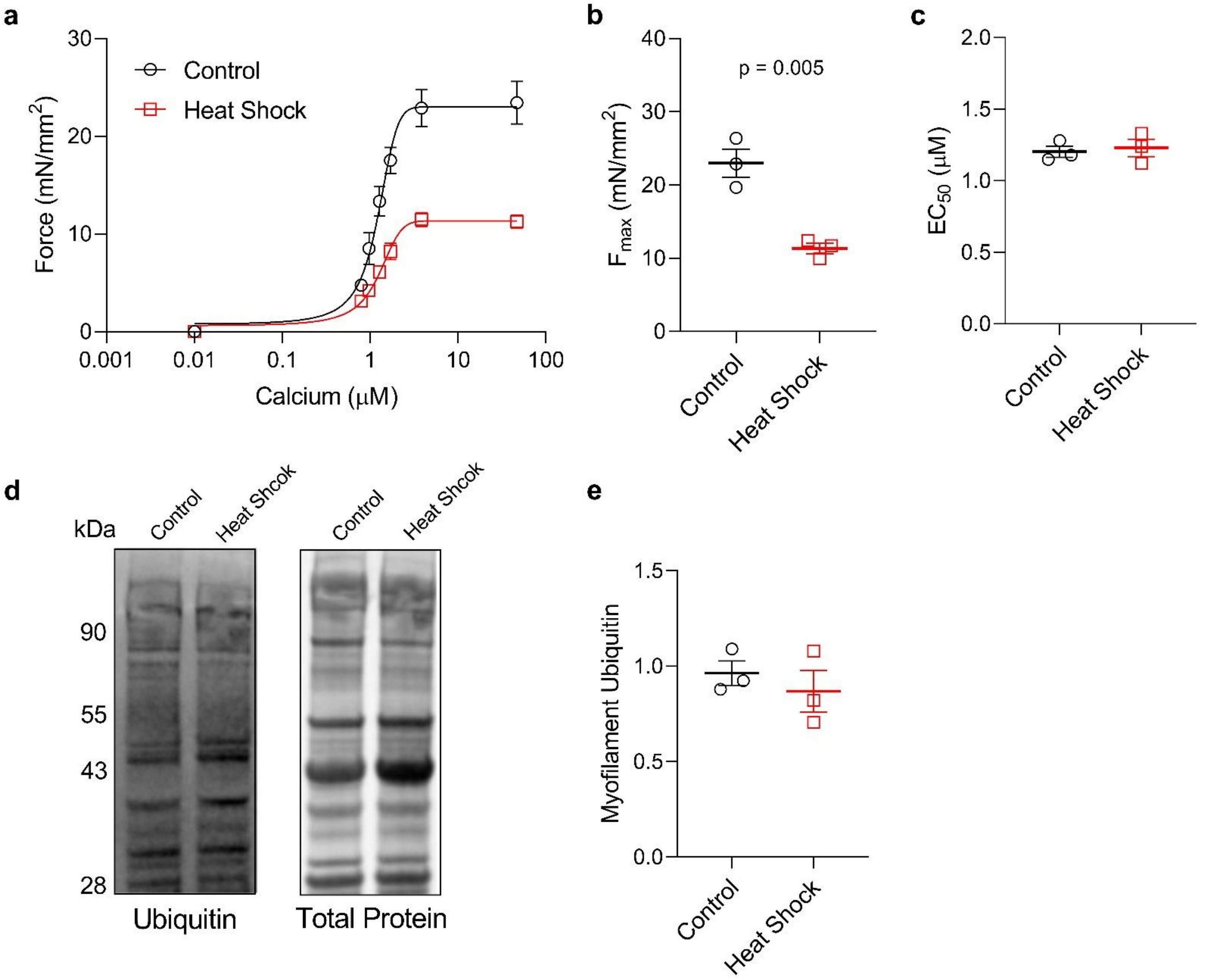

Supplementary Figure 1. Skinned human left ventricular cardiomyocytes display reduced F_{max} following heat shock | a, Force-calcium curves for skinned cardiomyocytes treated at 4 °C (Control) or at 43 °C (Heat Shock) for 3 hours; n = 3 myocytes/treatment from 3 separate non-failing patient samples. **b-c,** Summary data for individual myocyte F_{max} (b) and calcium sensitivity (c) corresponding to the force-calcium curves in a. **d,** Western blot for myofilament ubiquitin in the control and heat shock-treated samples; image is representative of 3 samples/treatment. **e,** Ubiquitin signal normalized to the total protein loading control; n = 3 samples/treatment. Data are presented as mean ± SEM and were analyzed by 2-tailed t-test.

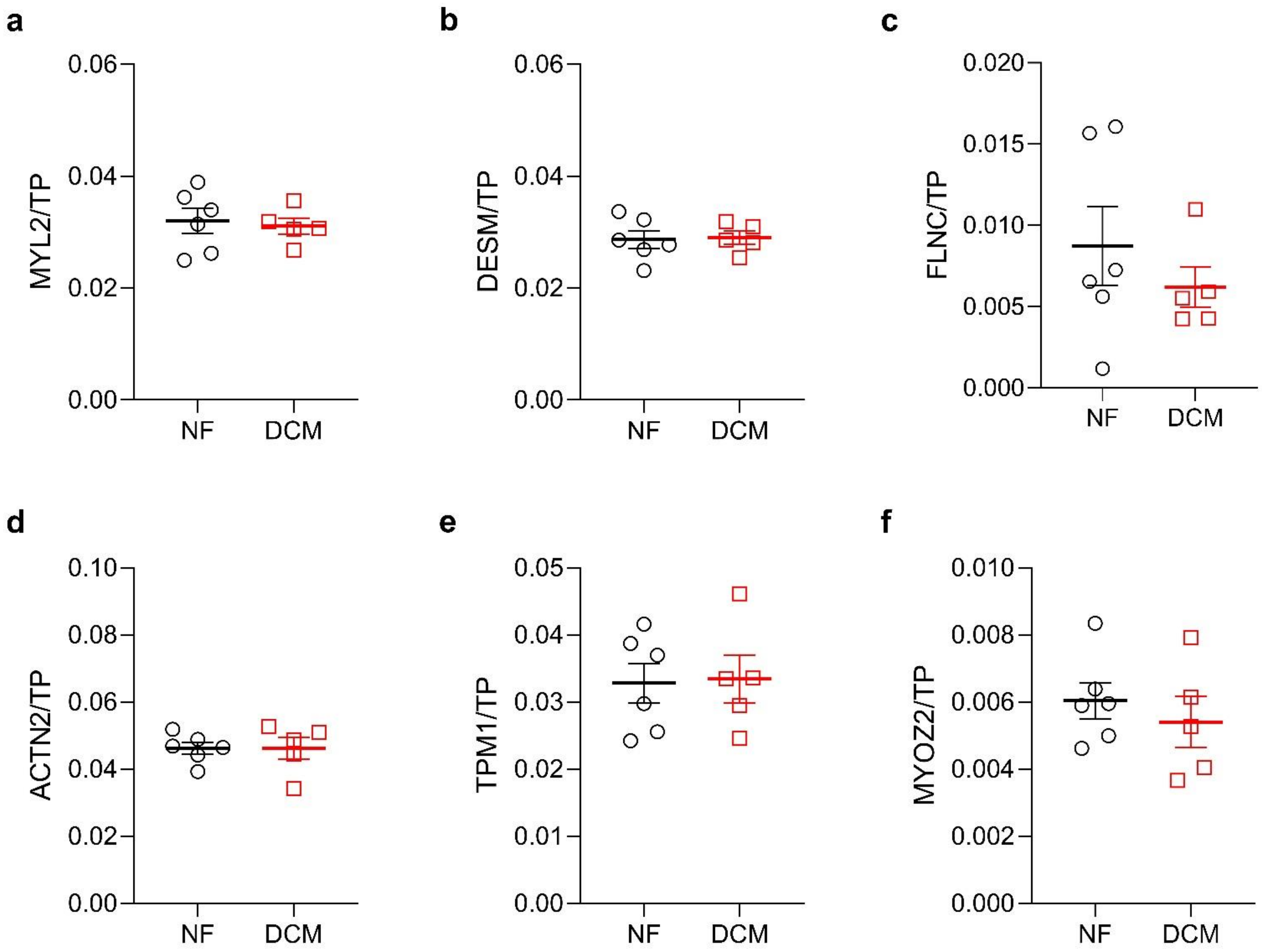

Supplementary Figure 2 | Total peptide amounts for the myofilament candidates with increased ubiquitination in human DCM are not different between the NF and DCM samples. **a-f,** Number of candidate peptides identified by LC-MS/MS normalized to the total peptide input for myosin regulatory light chain (a), desmin (b), filamin-C (c), α-actinin 2 (d), tropomyosin α-1 (e), and myozenin-2 (f). For all: n = 6 NF, 5 DCM. Data are presented as mean ± SEM.

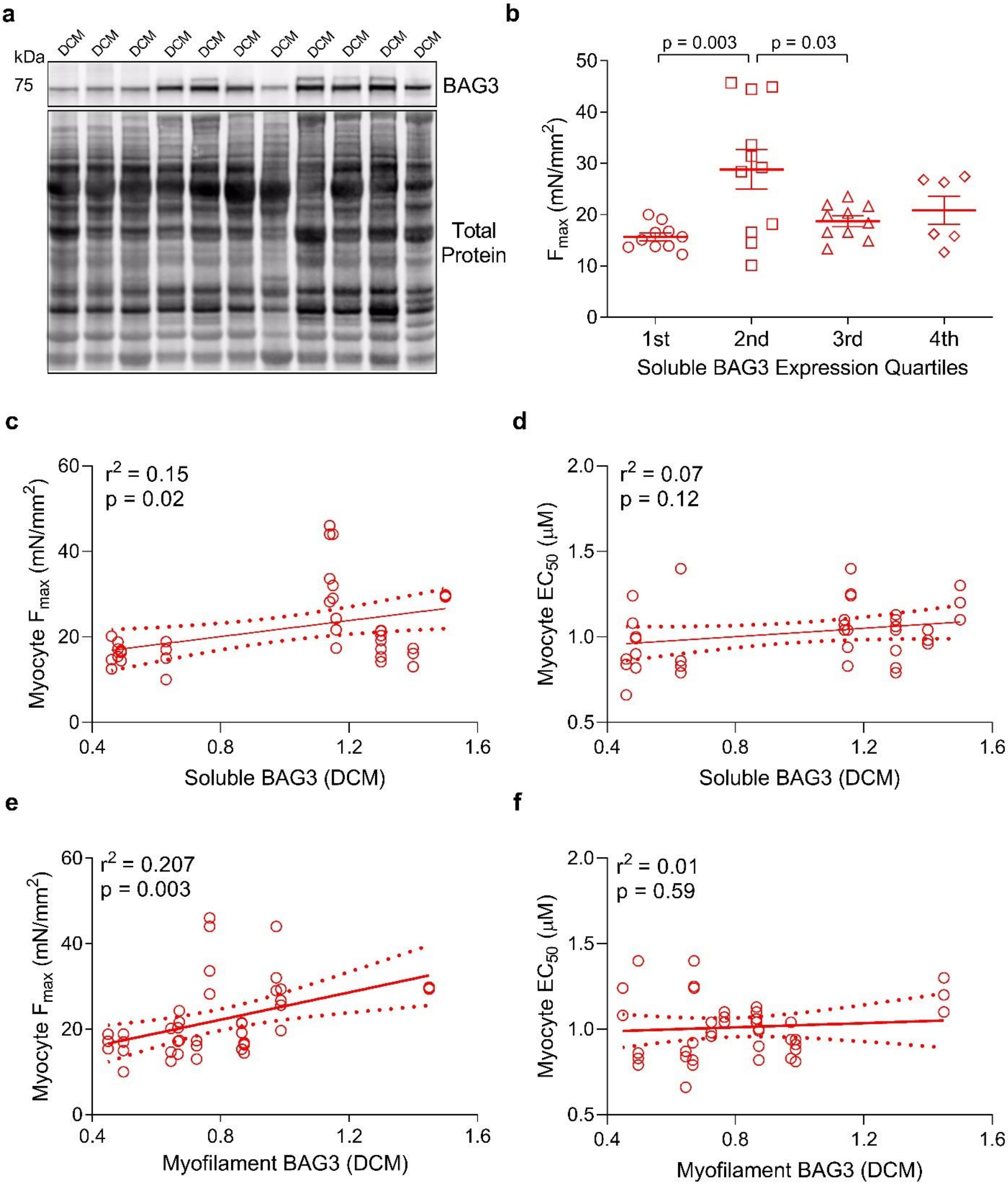

Supplementary Figure 3 | Soluble/cytosolic BAG3 expression does not correlate with myofilament functional parameters. a, Western blot for BAG3 in the soluble fraction from the DCM LV samples; image is representative of 11 DCM hearts. **b,** Myocyte F_{max} in the DCM samples organized by quartile of soluble BAG3 expression; 1st = lowest BAG3 expressors, 4th = highest; n = 11 DCM samples (one sample was not prepped for the soluble fraction due to low tissue amount), 3-4 myocytes per sample for functional assessment; Data are presented as mean ± SEM and were analyzed using one-way ANOVA, Tukey post-hoc. **c-d,** Linear regression comparison of cytosolic/soluble BAG3 expression with myocyte F_{max} (c) and EC_{50} (d). **e-f,** Linear regression comparison of myofilament BAG3 expression with myocyte F_{max} (e) and EC_{50} (f).

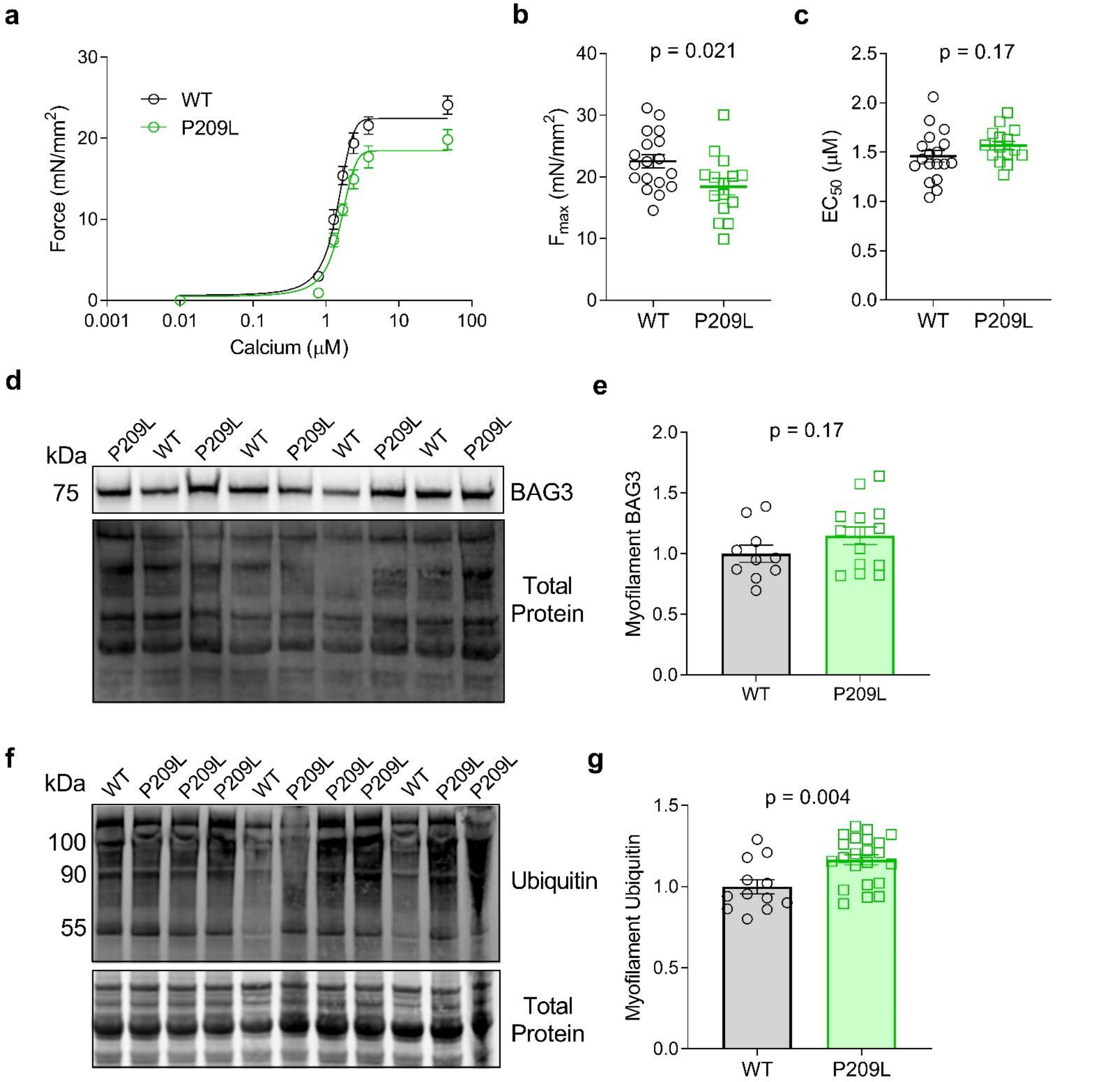

Supplementary Figure 4 | The P209L BAG3 mutation decreases sarcomere contractile force and impairs myofilament protein turnover. a, Skinned myocyte force-Ca^{2+} relationship for wild-type and P209L myocytes; n = 18 WT myocyte from 6 mice, 15 P209L from 5 mice. **b-c,** Summary data for individual myocyte F_{max} (b) and calcium sensitivity (c) corresponding to the force-calcium curves in a. **d,** Western blot for myofilament BAG3 in the WT and P209L mice; image is representative of 10 WT hearts and 14 P209L hearts. **e,** Myofilament BAG3 expression normalized to total protein; n = 10 WT, 14 P209L. **f,** Western blot for myofilament ubiquitin in the WT and P209L mice; image is representative of 12 WT hearts and 22 P209L hearts. **g,** Myofilament ubiquitin expression normalized to total protein; n = 12 WT, 22 P209L. All data are presented as mean ± SEM and were analyzed by 2-tailed t-test.

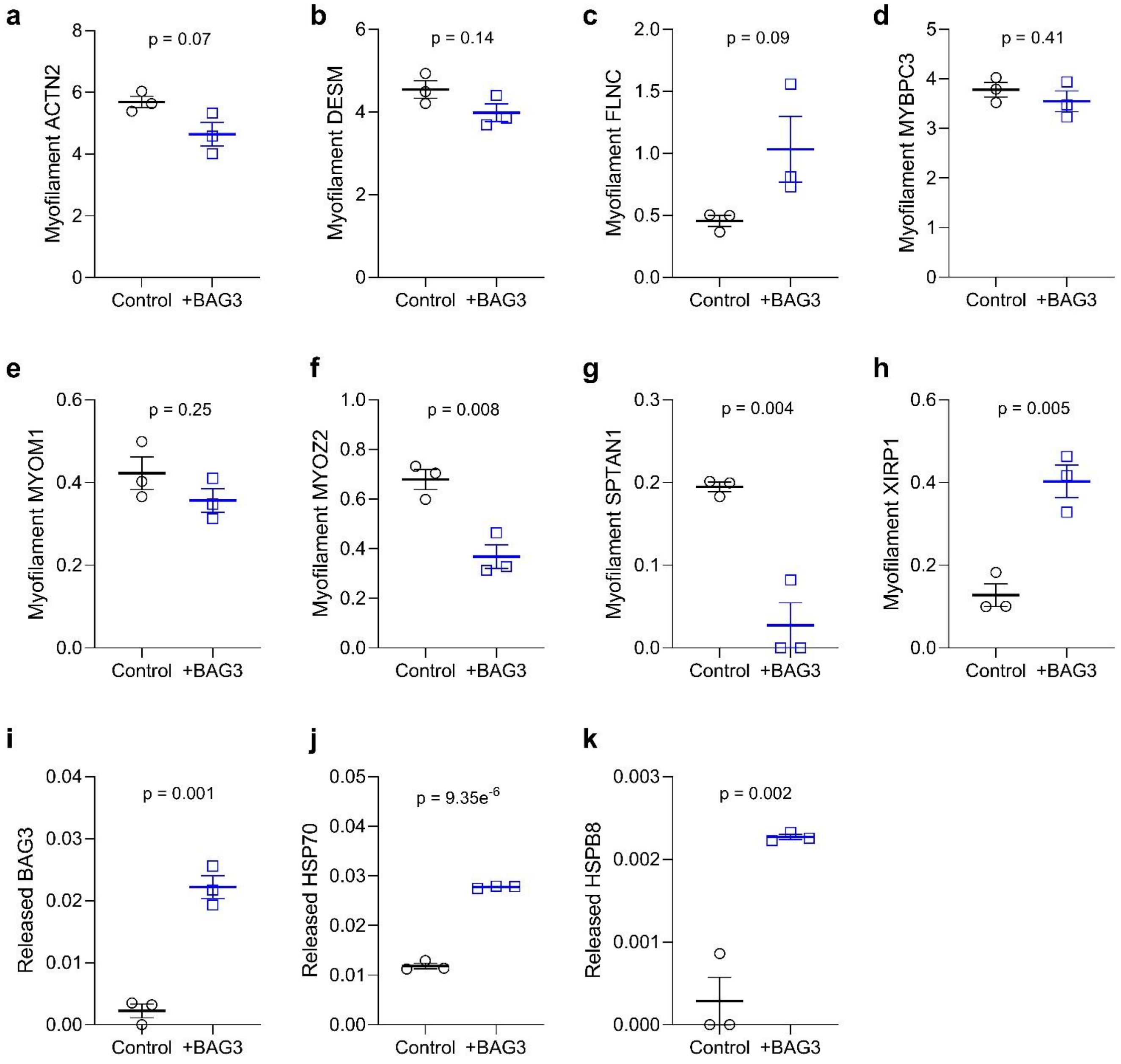

Supplementary Figure 5 | Mass spectrometry results of the myofilament pool of CASA clients and the released CASA complex members from the client release experiment. a-h, Analysis of the 8 CASA clients in the myofilament/insoluble protein fraction of three DCM human samples after 1 hour of treatment with control or 3 µM recombinant BAG3 protein. **i-k,** Analysis of BAG3 (i), HSP70 (j), and HSPB8 (k) in the released fraction, indicating that HSP70 and HSPB8 are also released from the myofilament with increased BAG3. For all: n = 3 control, 3 +BAG3 from the sample 3 human LV tissue samples. All data are presented as the mean ± SEM and were analyzed with 2-tailed t-test.

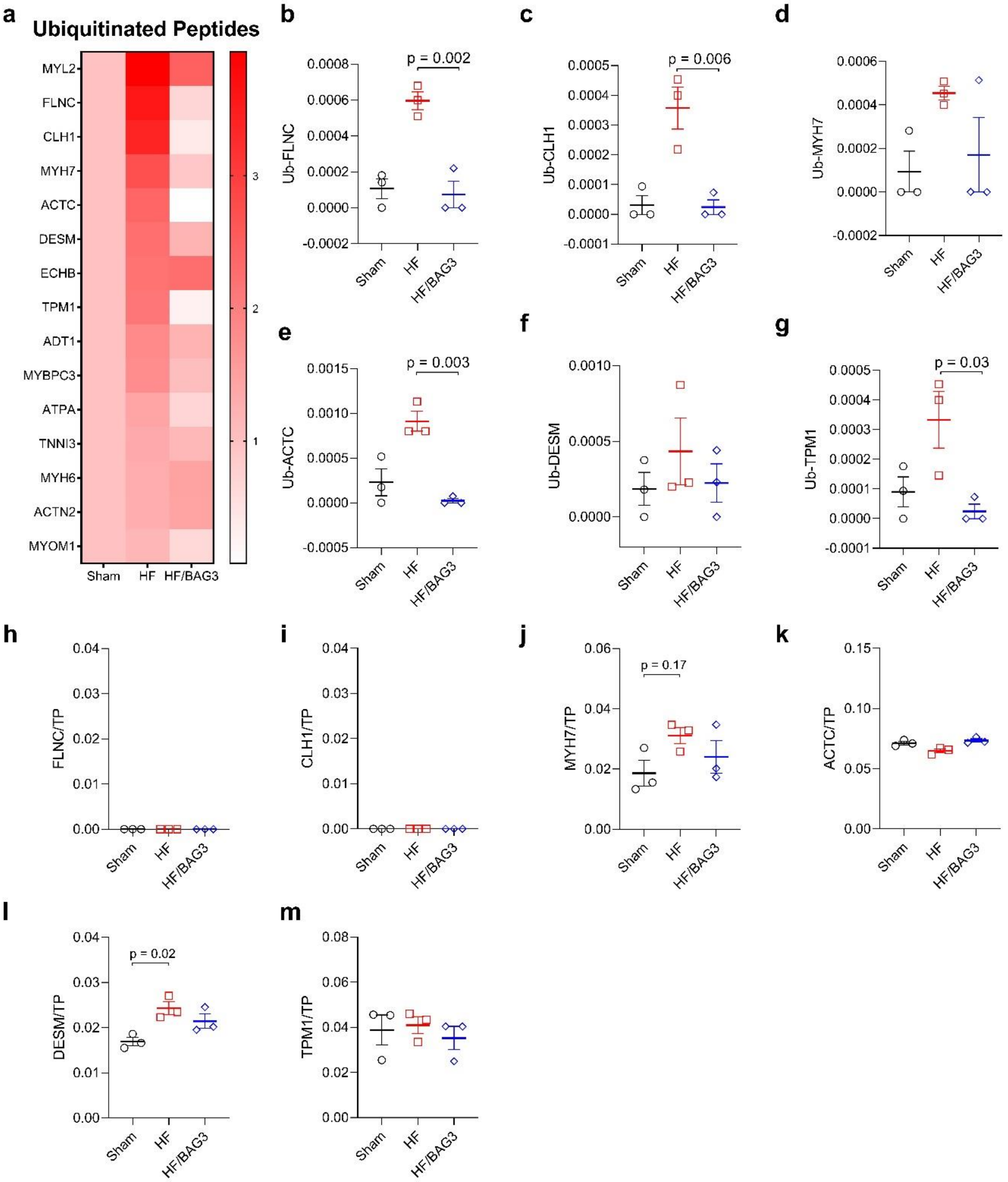

Supplementary Figure 6 | De novo ubiquitinated peptide data for the mouse heart failure model with BAG3 gene therapy. a, Heat map of the top 15 most changed ubiquitinated proteins from the Sham, HF, and HF/BAG3 ubiquitinated de novo peptides identified by mass spectrometry; scale bar = fold change increase relative to Sham; darkest red denotes greatest fold increase. **b-g,** Spectral count analysis of potential candidates for BAG3/CASA-mediated protein turnover (FLNC- filamin C, CLH1 – clathrin heavy chain 1, MYH7 – myosin heavy chain 7, ACTC – cardiac alpha actin, DESM – desmin, TPM1 – tropomyosin alpha 1); the number of ubiquitinated de novo peptides were normalized to total peptide controls separated prior to ubiquitin enrichment. **h-m,** Spectral count data of the relative amount of candidate peptides in the total peptide input showing that peptide amounts were unchanged between groups, except for DESM, which increased in the HF group. For all n = 3 samples per group, data are presented as the mean ± SEM and were analyzed by one-way ANOVA with Tukey's post-hoc test for multiple comparisons.

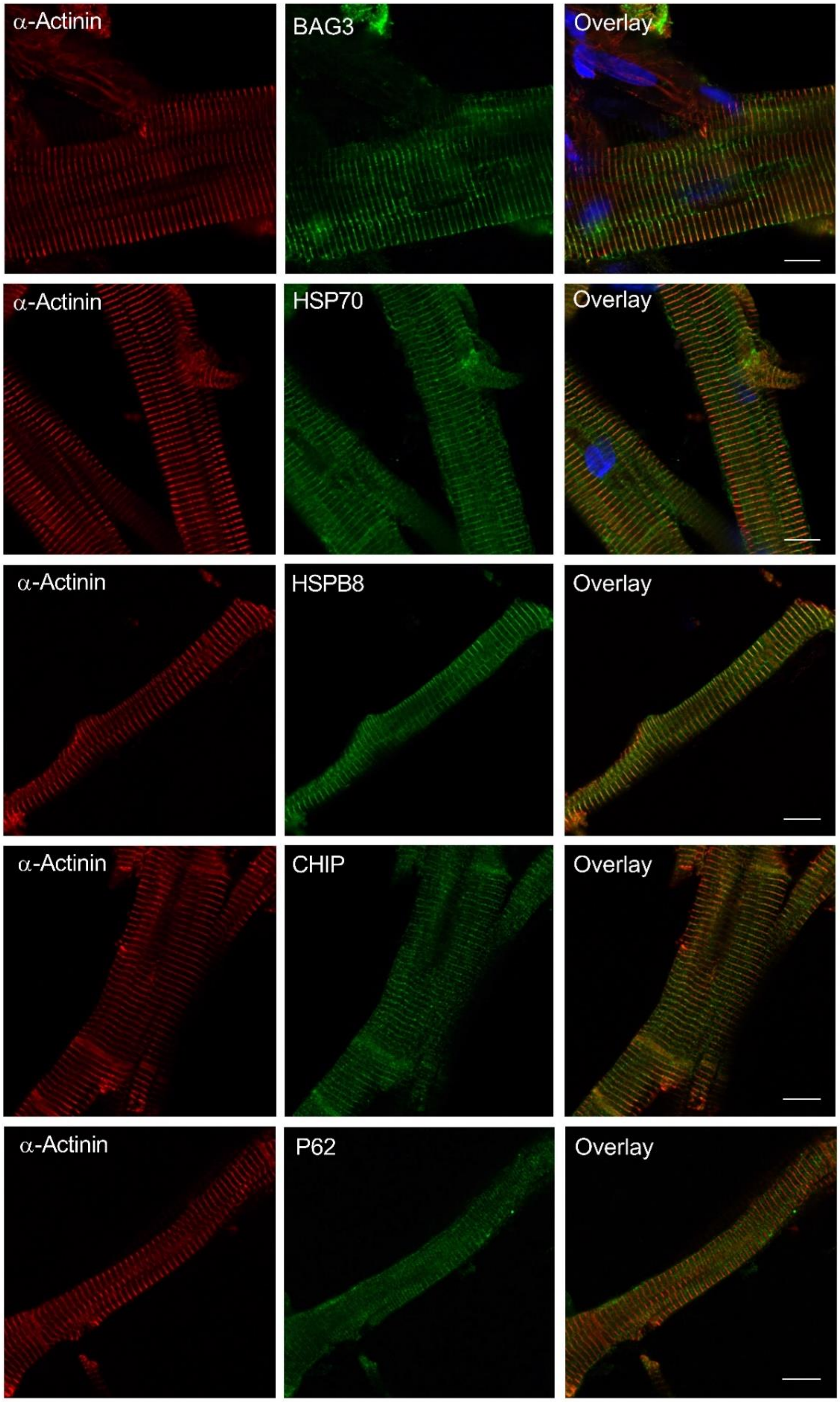

Supplementary Figure 7 | Uncropped immunofluorescence images from the human left ventricle cardiomyocytes. All images were acquired at 63X magnification; scale bars represent 10 microns; images are representative 10 images/antibody/5 independent biological samples.

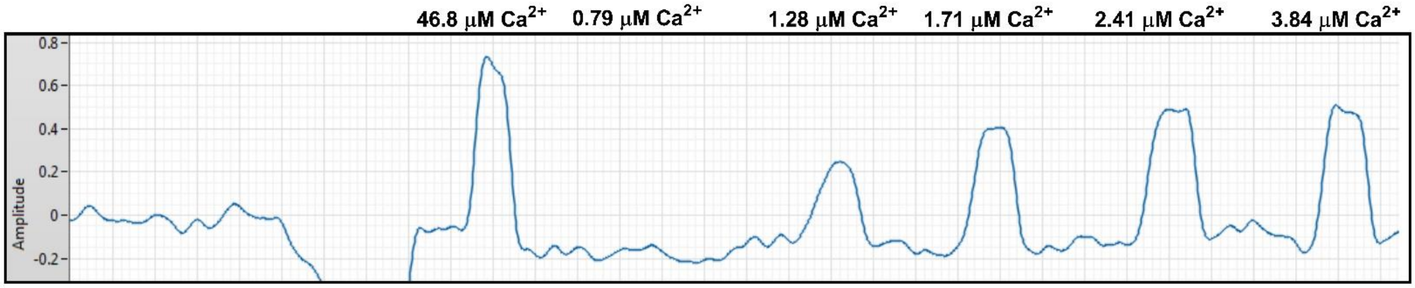

Supplementary Figure 8 | Example recording from the skinned myocyte force-calcium experiments. Single myocyte contractile force in response to various concentrations of calcium. Y-axis amplitude = millivolts; converted to mN using equation derived from force transducer calibration. Contractile forces were normalized to myocyte cross-sectional area during analysis.

Supplementary Figure 9. UNCROPPED WESTERN BLOTS

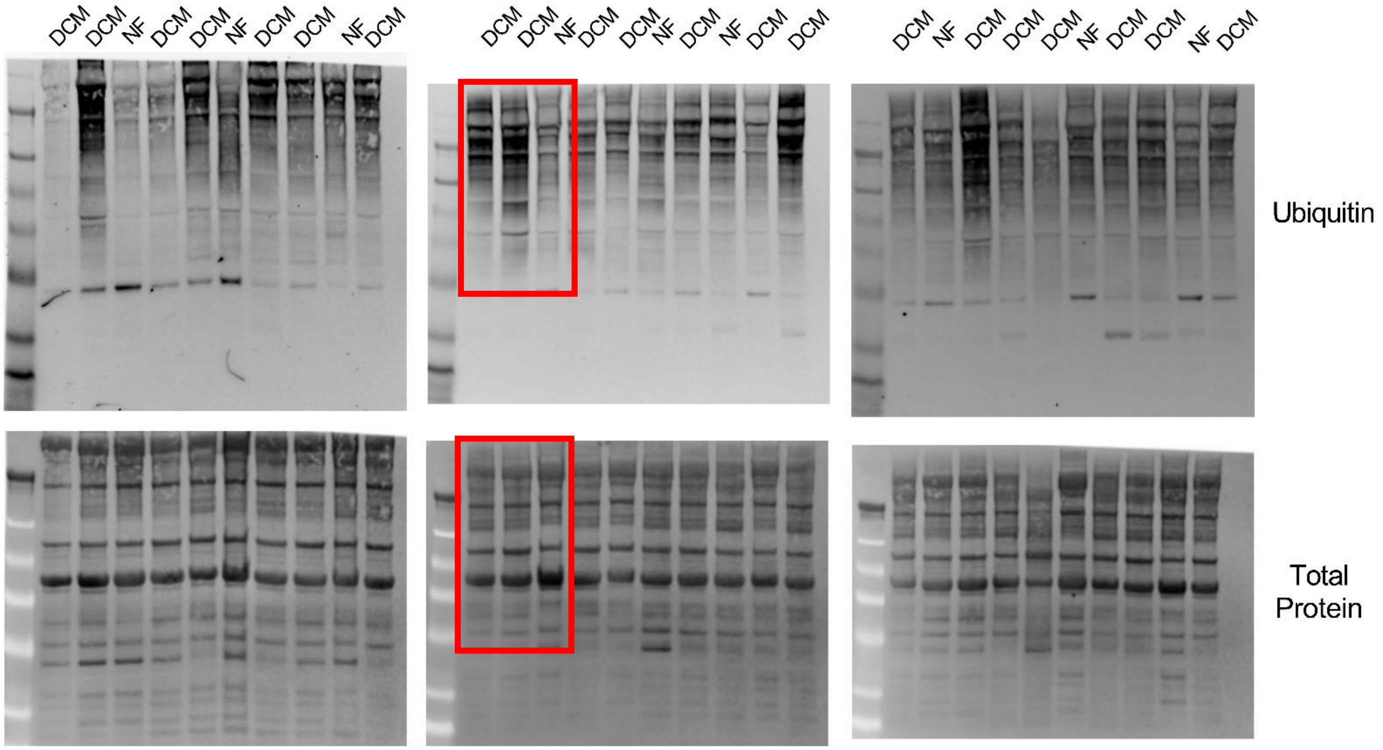

Uncropped western blots pertaining to Figure 1D for ubiquitin in myofilament-enriched human non-failing (NF) and dilated cardiomyopathy (DCM) samples.

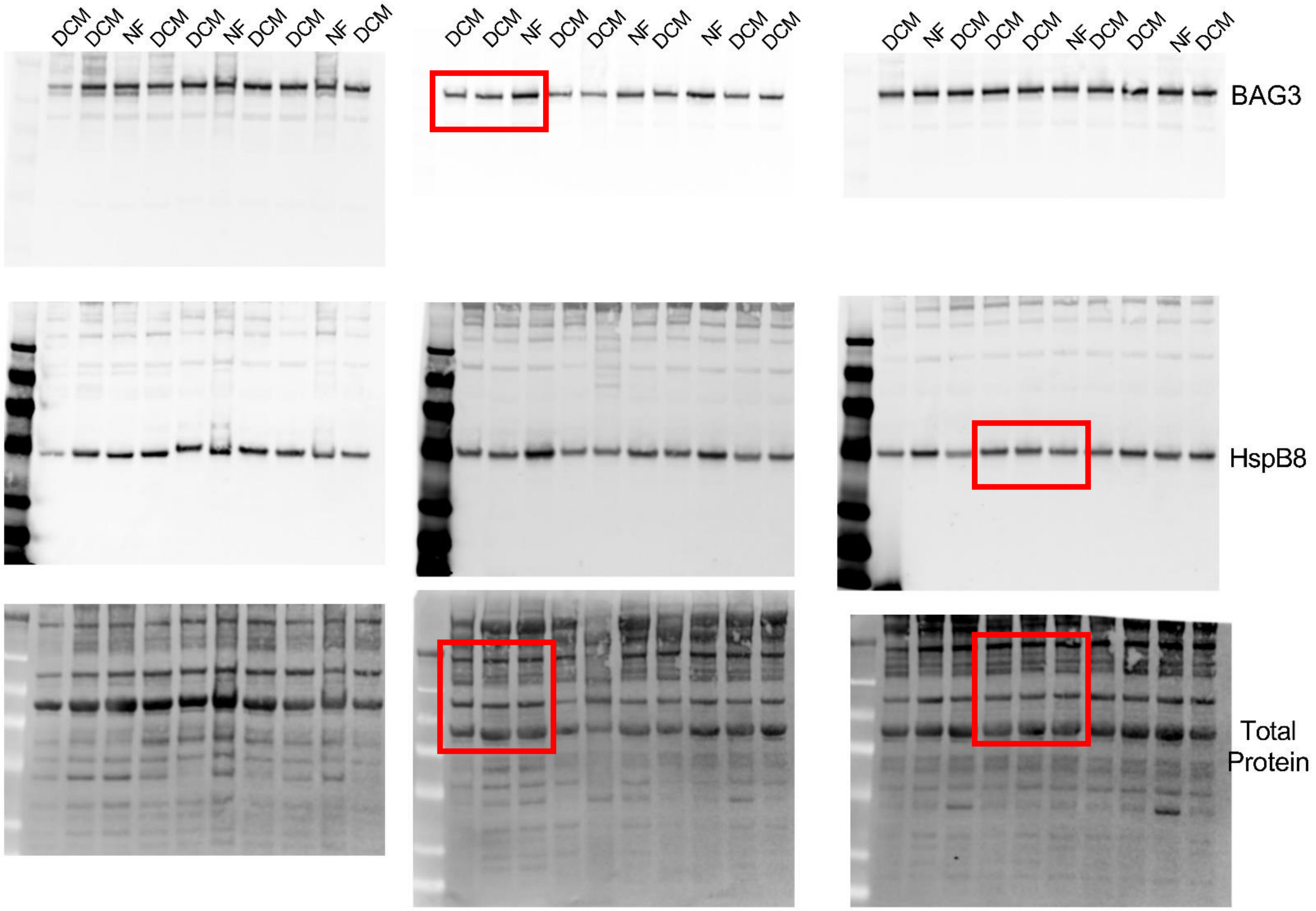

Uncropped western blots for BAG3 and HspB8 in myofilament-enriched human non-failing (NF) and dilated cardiomyopathy (DCM) samples pertaining to Figures 2A and 5G.

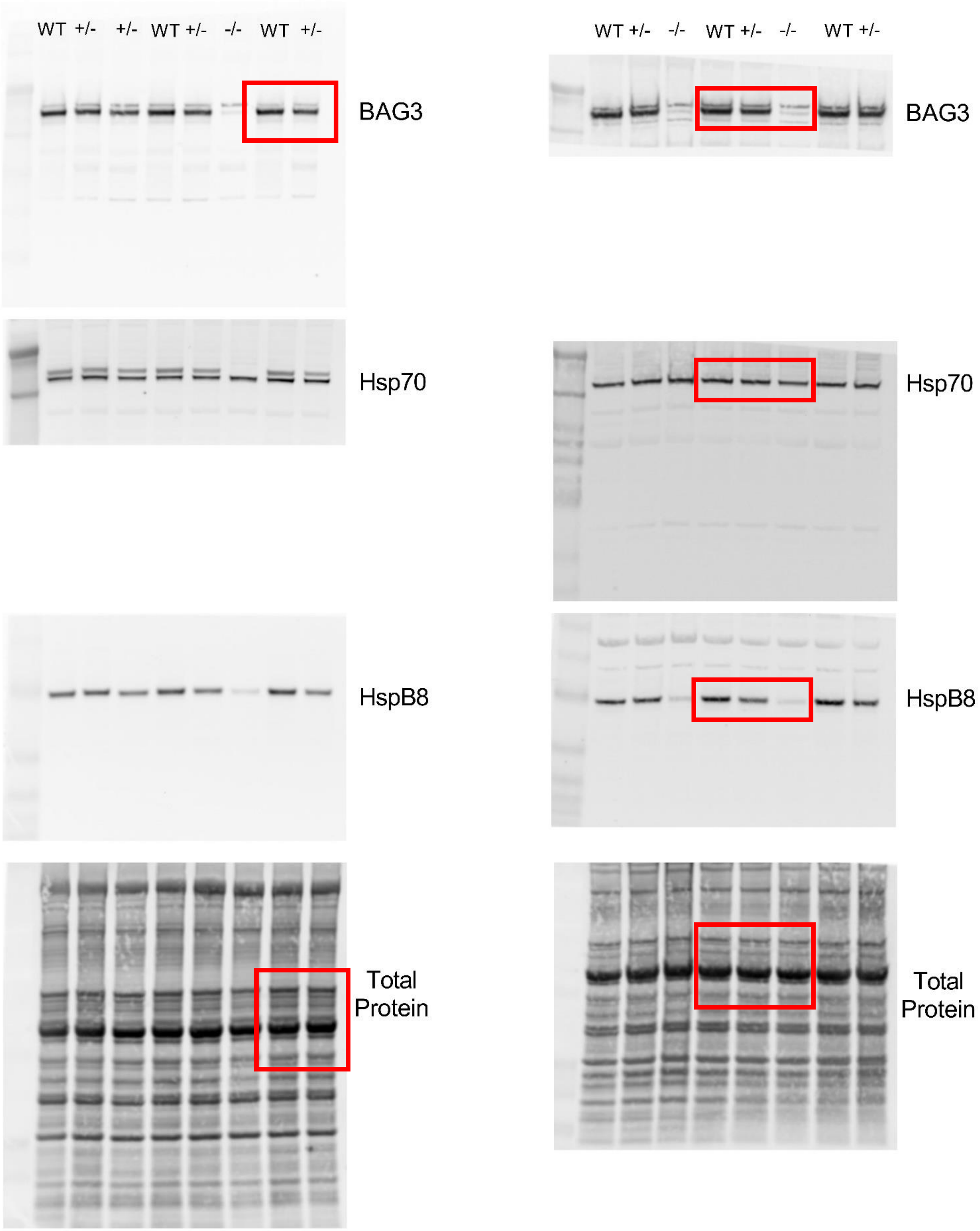

Uncropped western blots for BAG3, Hsp70, and HspB8 in myofilament-enriched tissue from the 6-week-old WT, BAG3 +/-, and BAG3 -/- mice pertaining to Figures 2E-F and 5A-C.

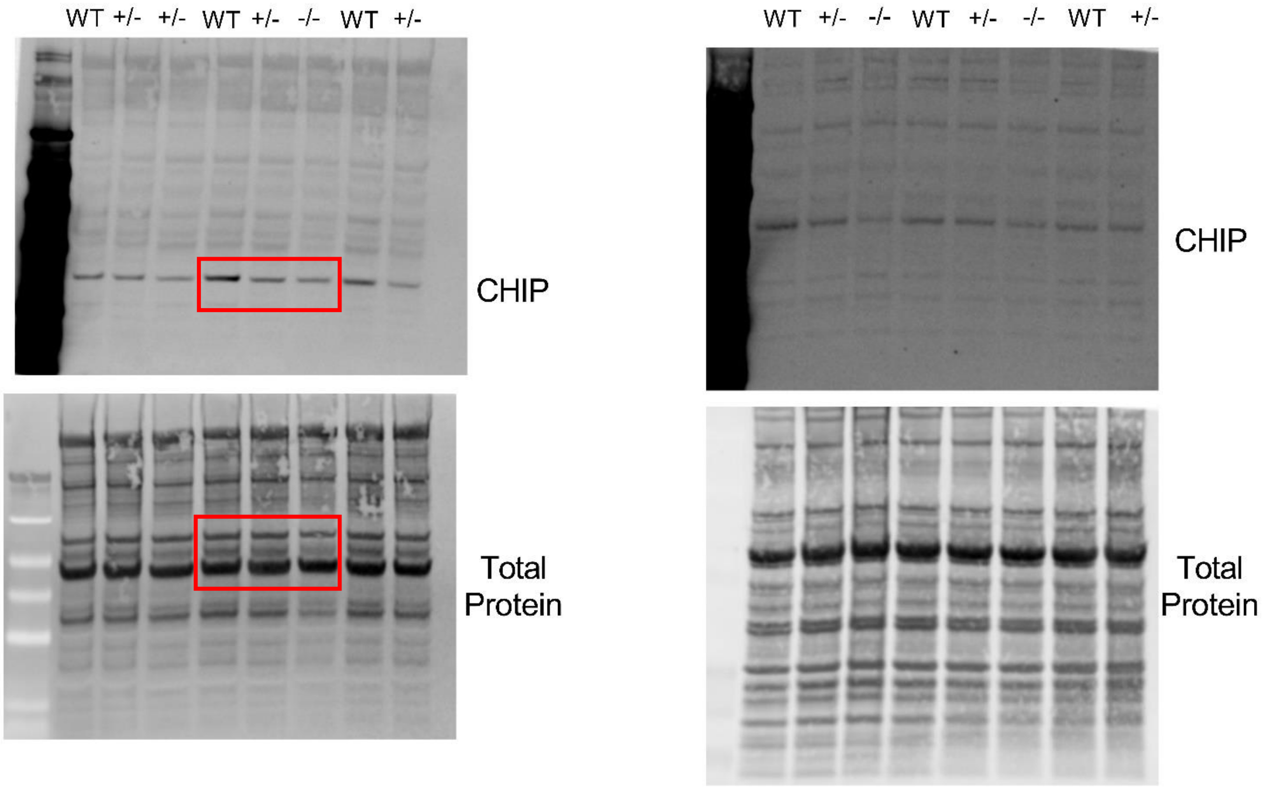

Uncropped western blots for CHIP in myofilament-enriched tissue from the 6-week-old WT, BAG3 +/, and BAG3 -/- mice pertaining to Figures 5A & 5D.

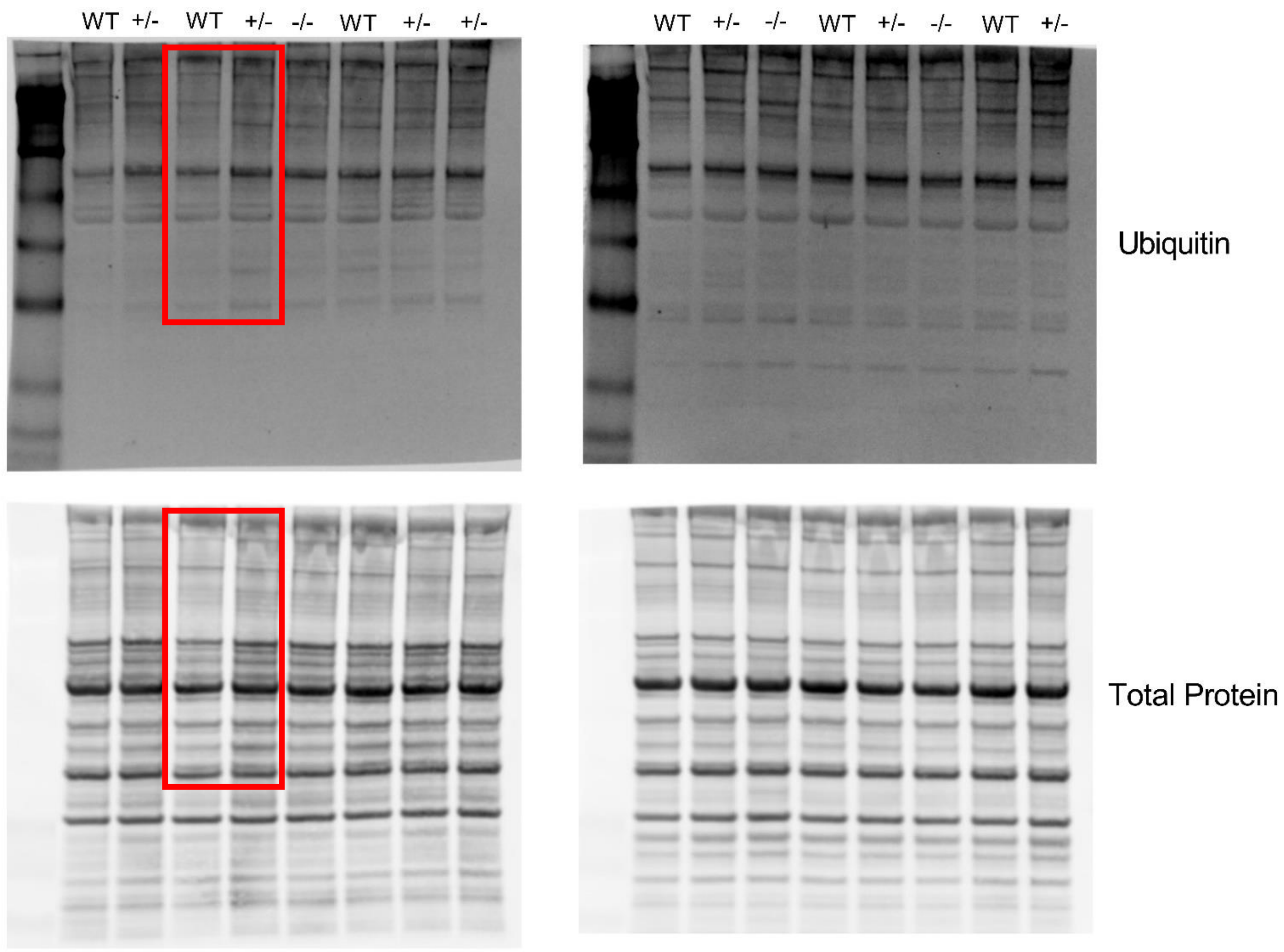

Uncropped western blots for ubiquitin in myofilament-enriched tissue from the 6-week-old WT, BAG3 +/-, and BAG3 -/- mice pertaining to Figure 2G-H.

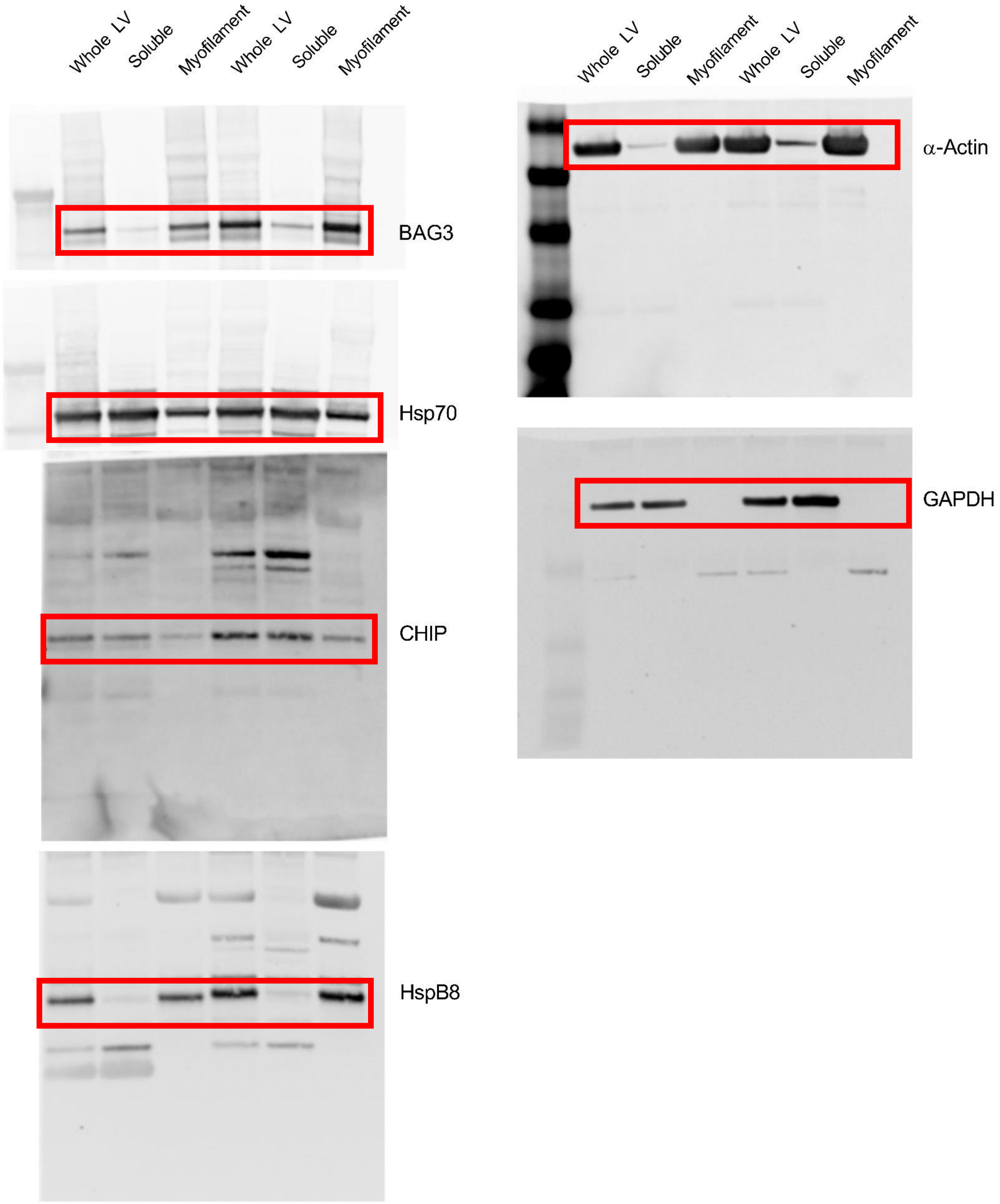

Uncropped western blots for the CASA complex proteins in myofilament-enriched human LV tissue pertaining to Figure 3C.

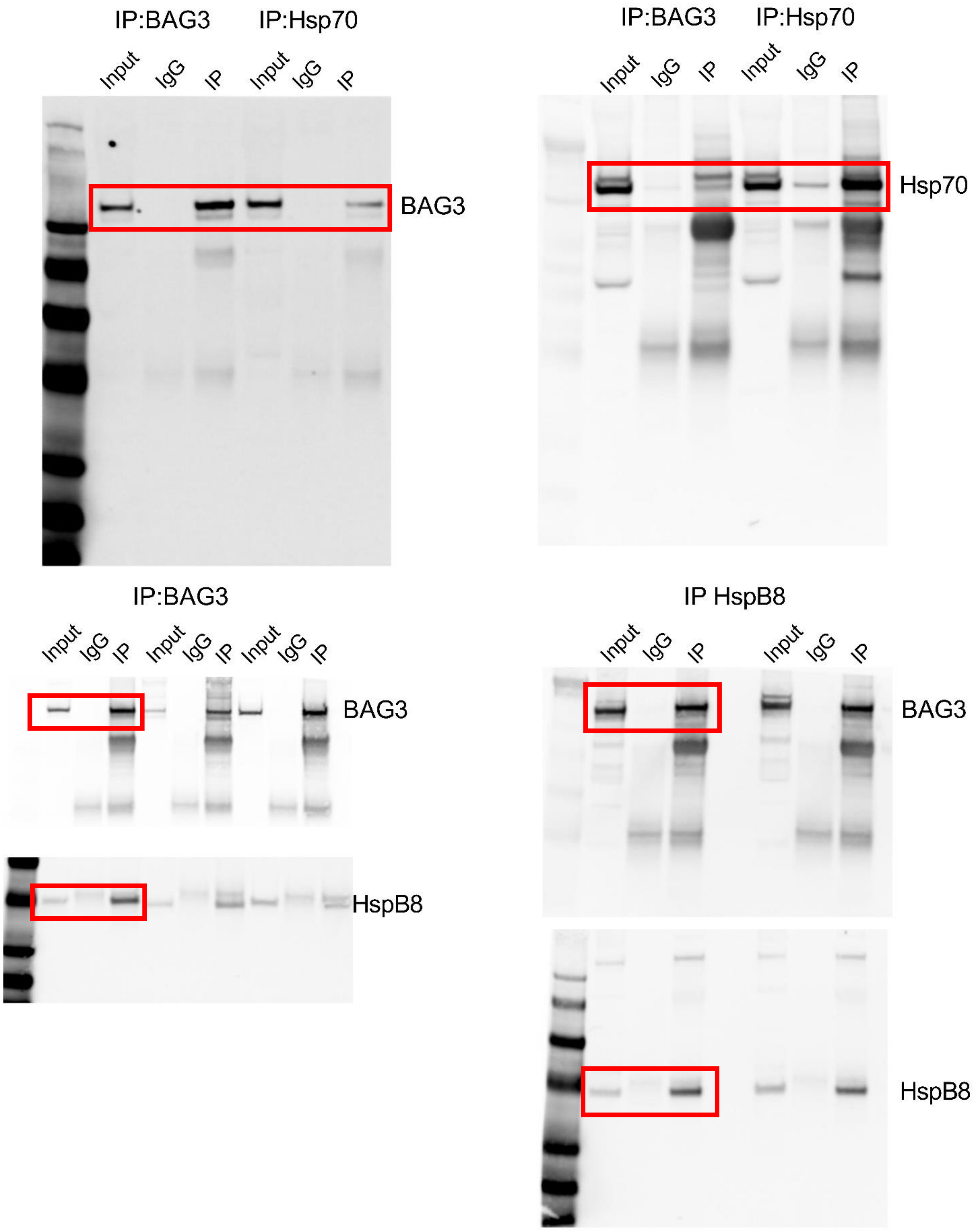

Uncropped western blots for the co-immunoprecipitation experiments for BAG3 with Hsp70 and HspB8 in human LV myofilament-enriched fractions pertaining to Figure 3D-E.

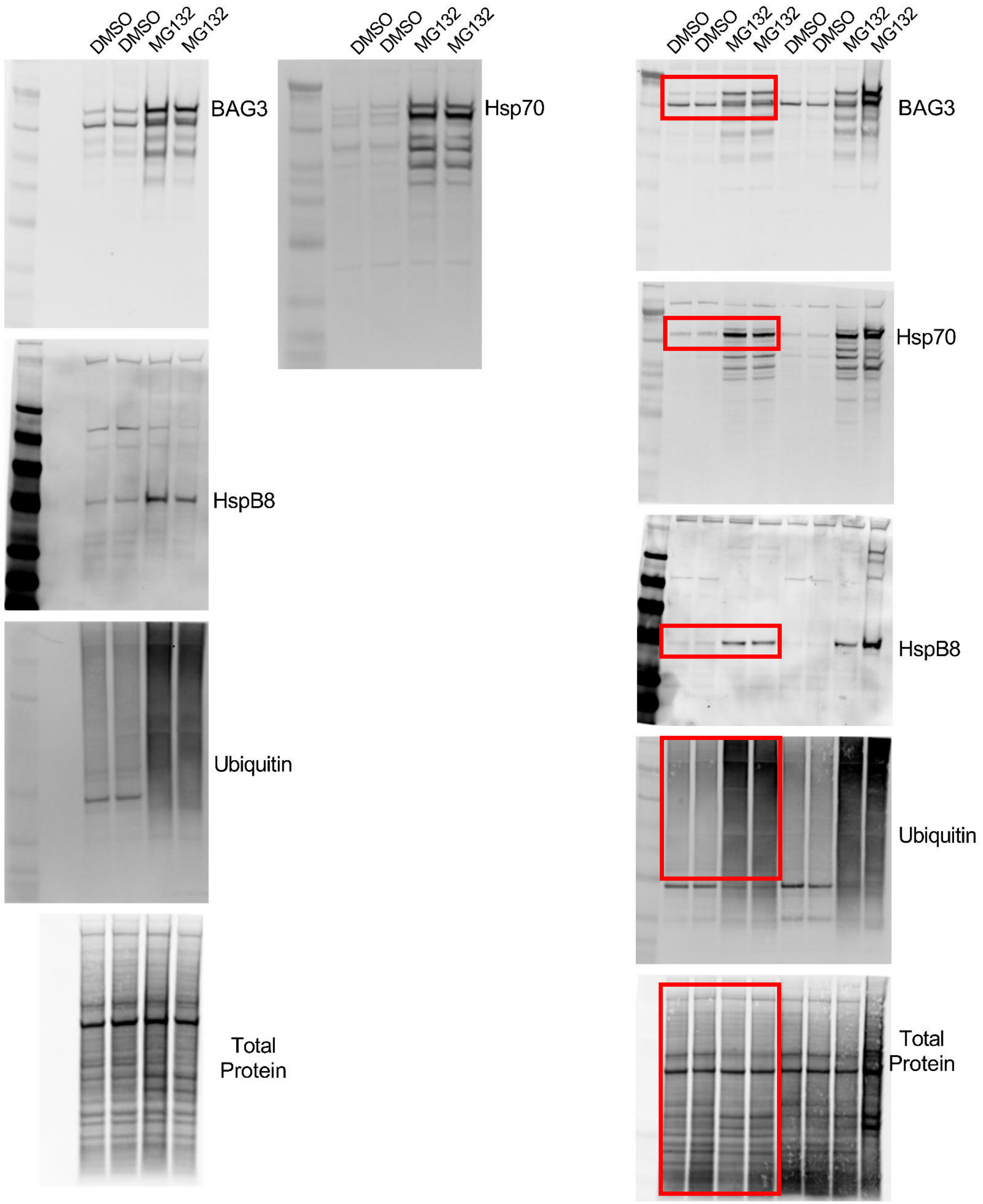

Uncropped western blots for myofilament BAG3, HspB8, Hsp70, and Ubiquitin in the NRVM experiments pertaining to Figure 4A-G.

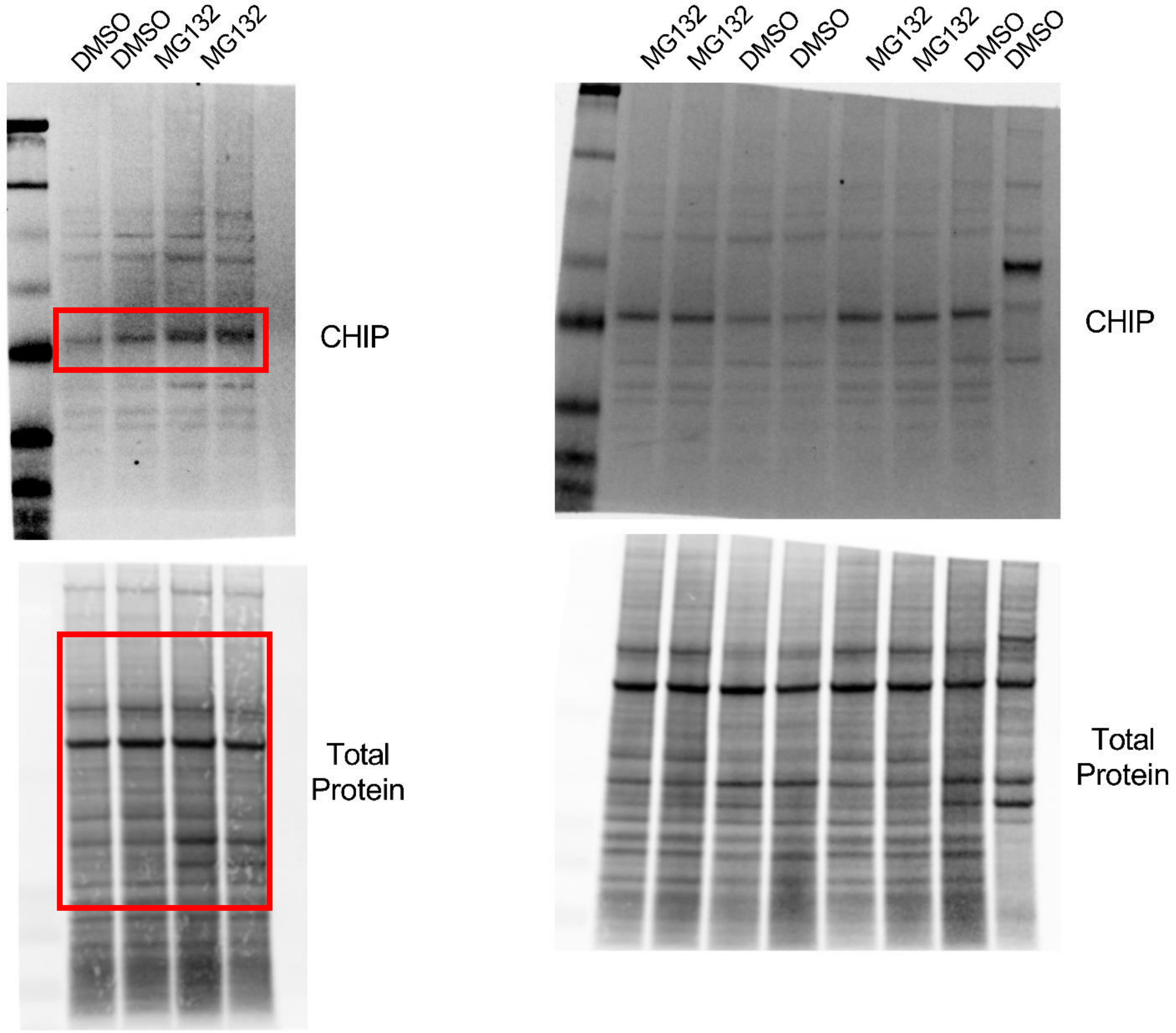

Uncropped western blots for myofilament CHIP in the NRVM experiments pertaining to Figure 4B & 4F.

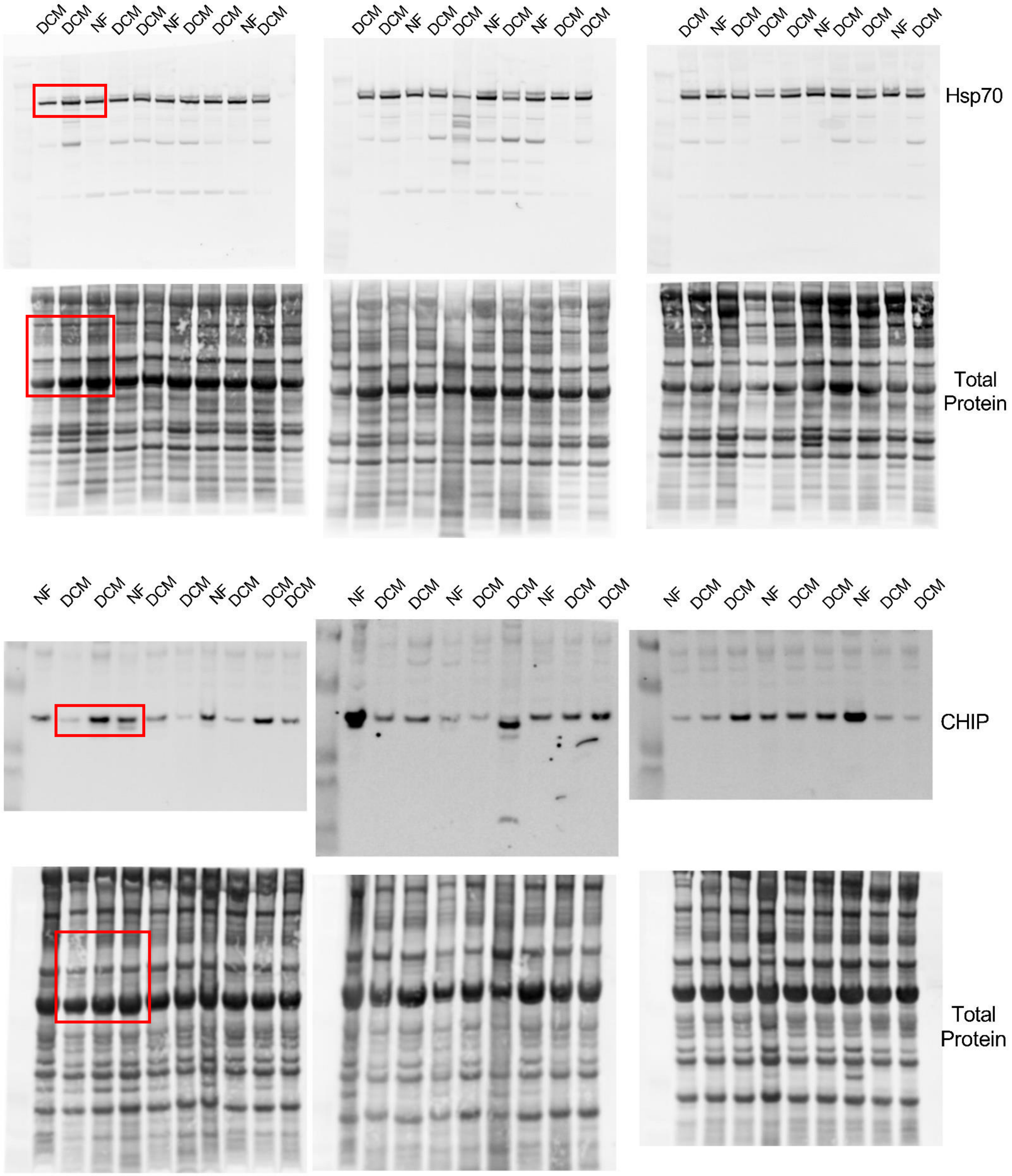

Uncropped western blots for Hsp70 and CHIP in myofilament-enriched human non-failing (NF) and dilated cardiomyopathy (DCM) samples pertaining to Figure 5E-J.

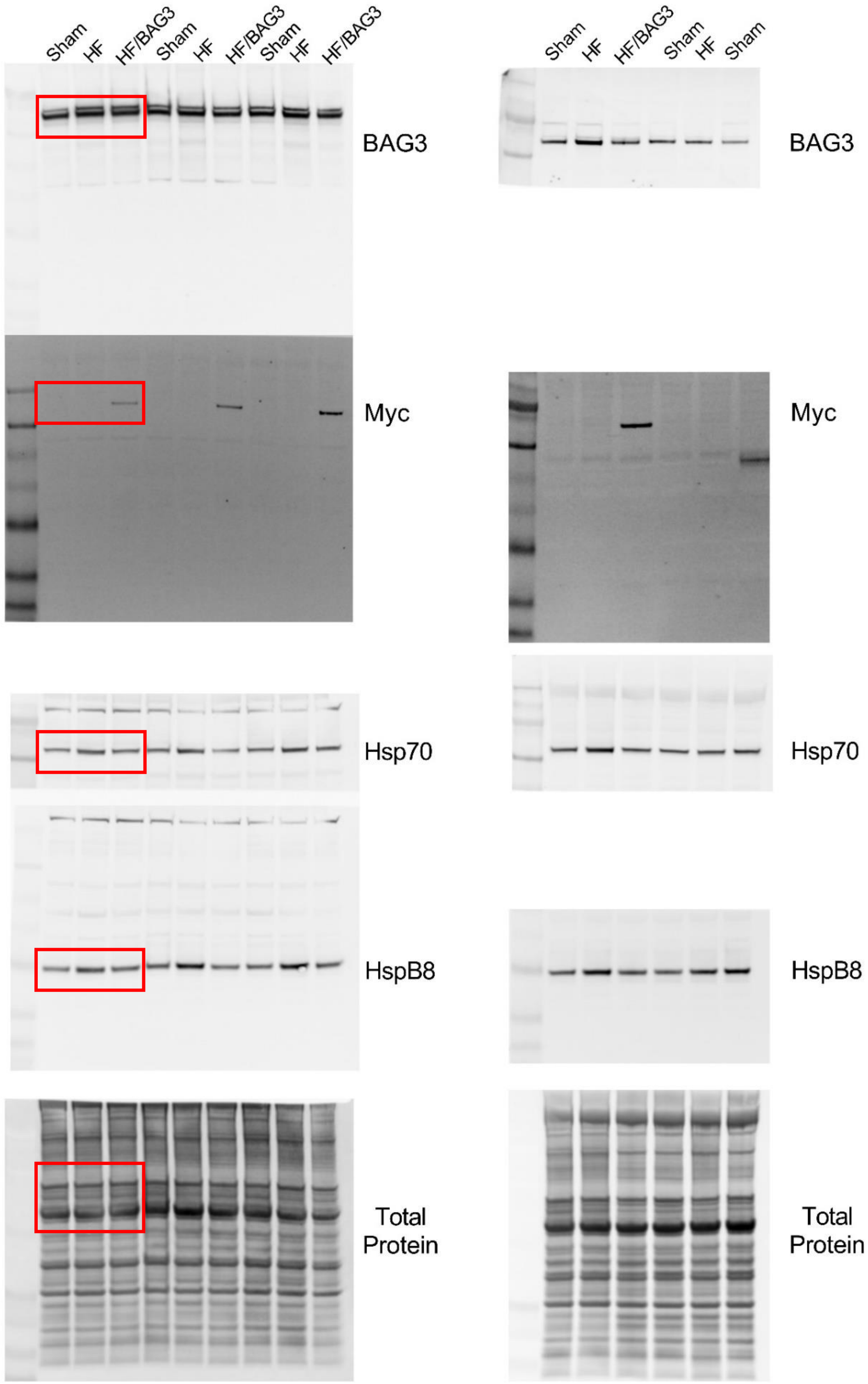

Uncropped western blots for myofilament-specific BAG3, Myc, Hsp70, and HspB8 in the mouse HF model treated with BAG3 gene therapy pertaining to Figure 7A-E.

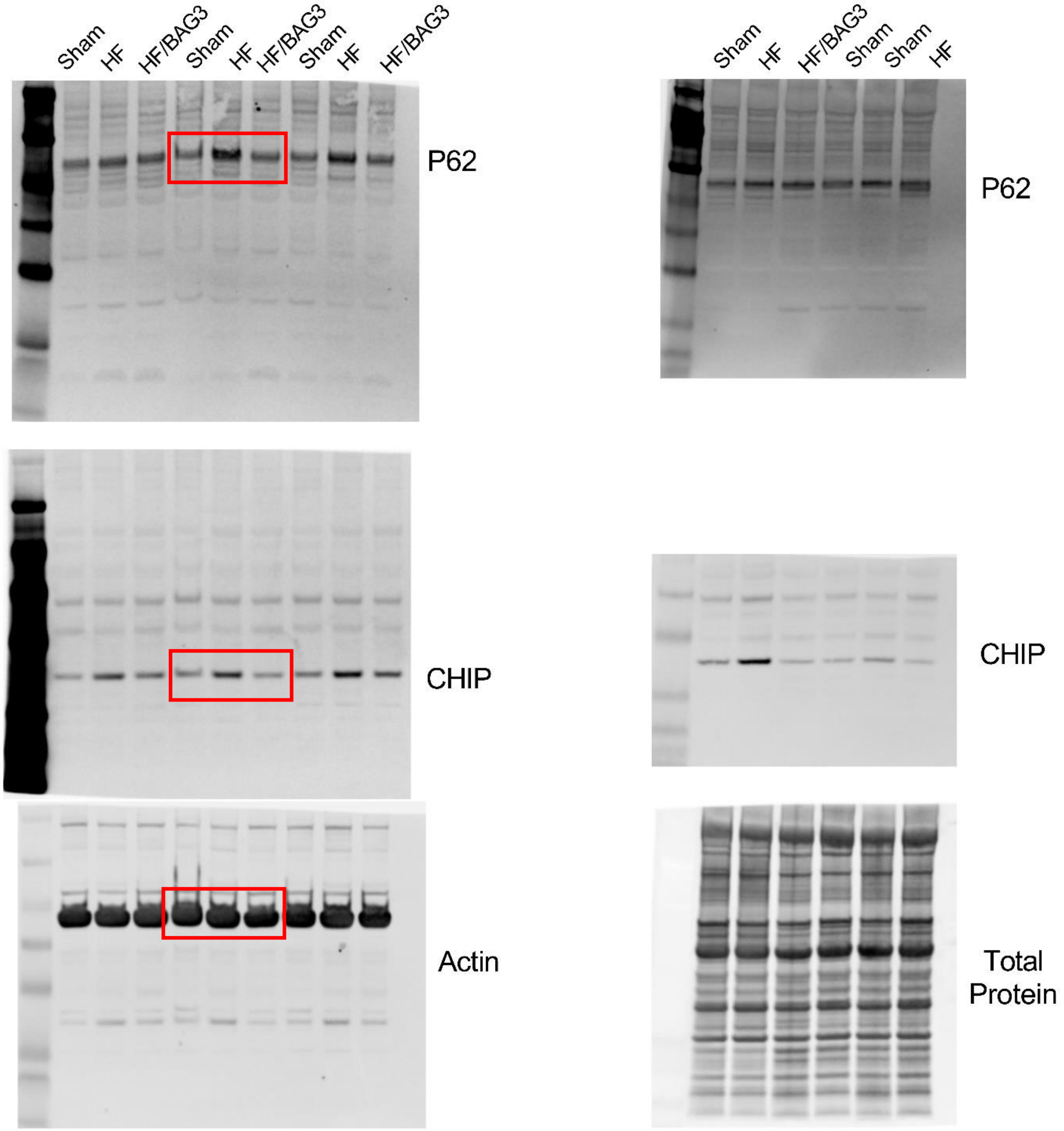

Uncropped western blots for myofilament-specific P62 and CHIP in the mouse HF model treated with BAG3 gene therapy pertaining to Figure 7F-H.

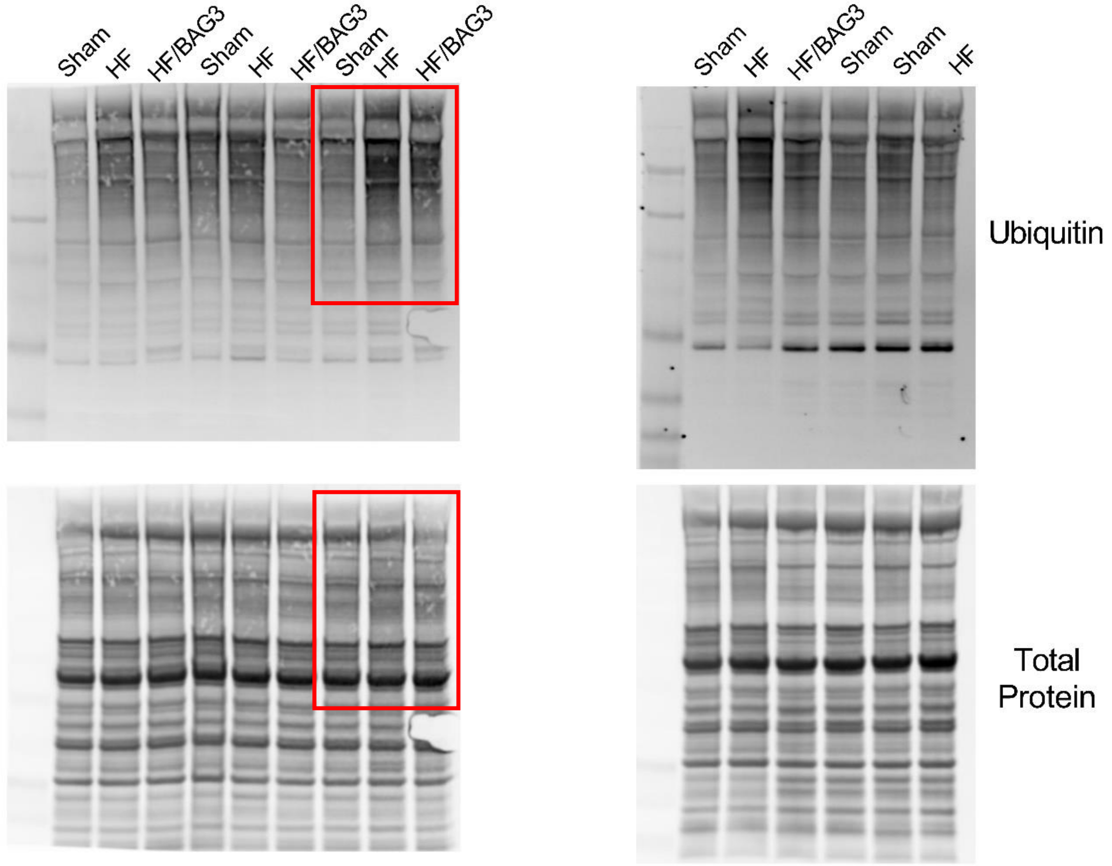

Uncropped western blots for myofilament-specific ubiquitin in the mouse HF model treated with BAG3 gene therapy pertaining to Figure 6E-F.

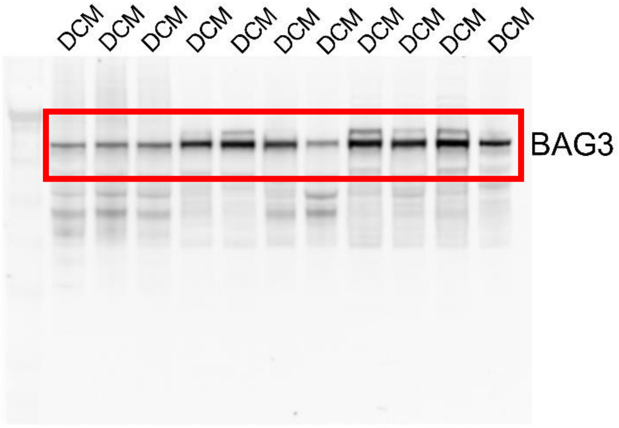

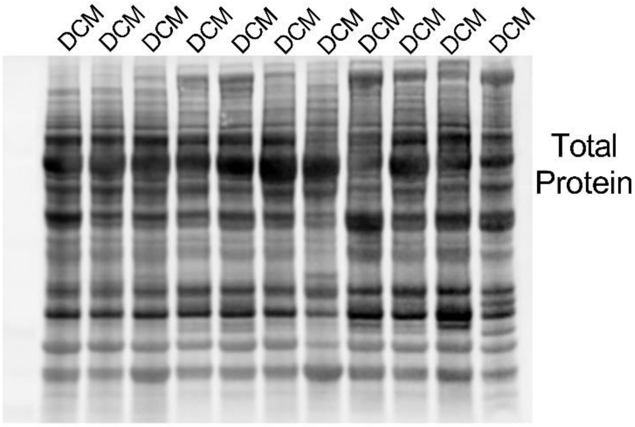

Uncropped western blots for BAG3 in the cytosolic/triton-soluble fraction of human DCM LV samples pertaining to Supplementary Figure 3.

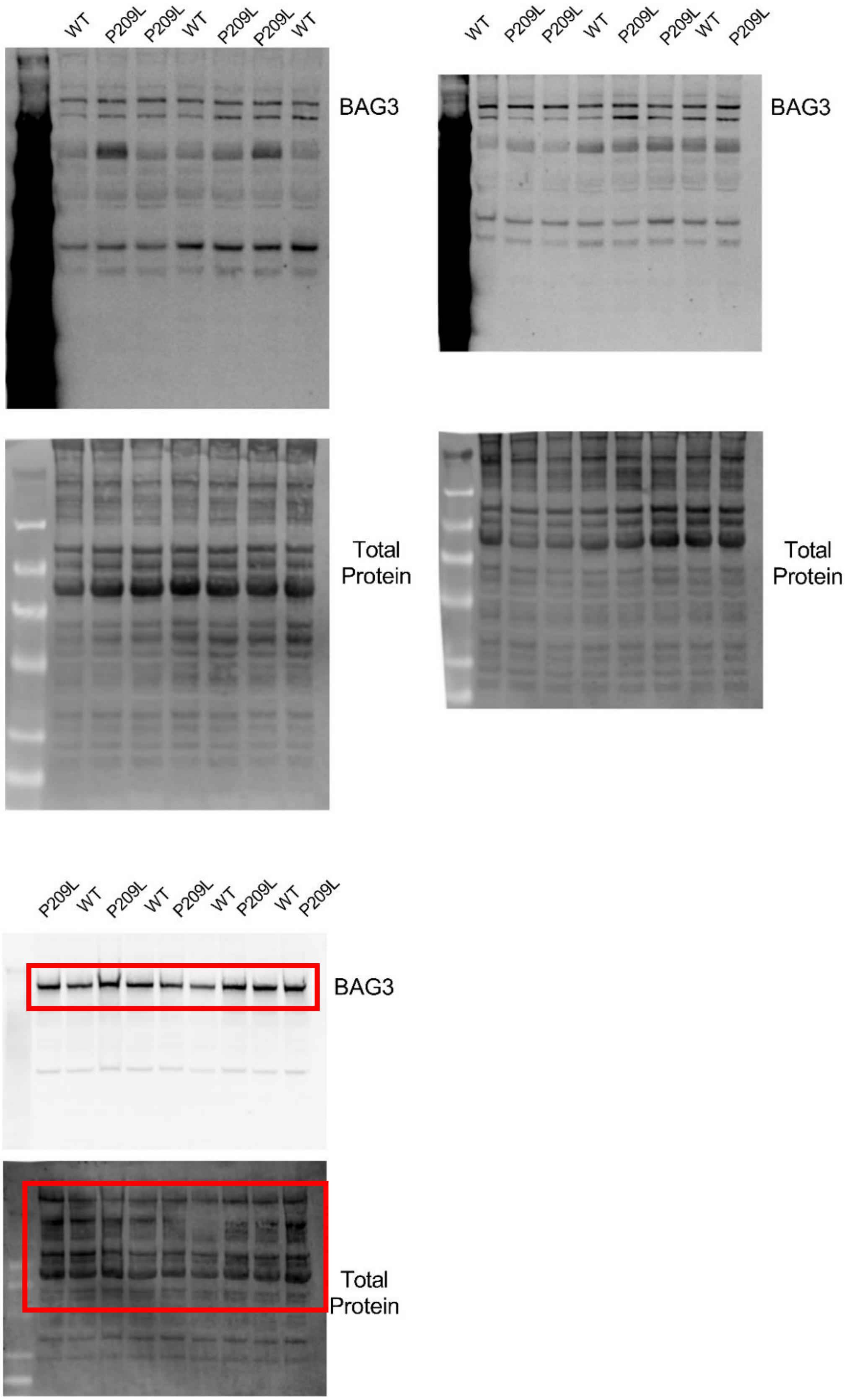

Uncropped western blots for BAG3 in the P209L mice pertaining to Supplementary Figure 4.

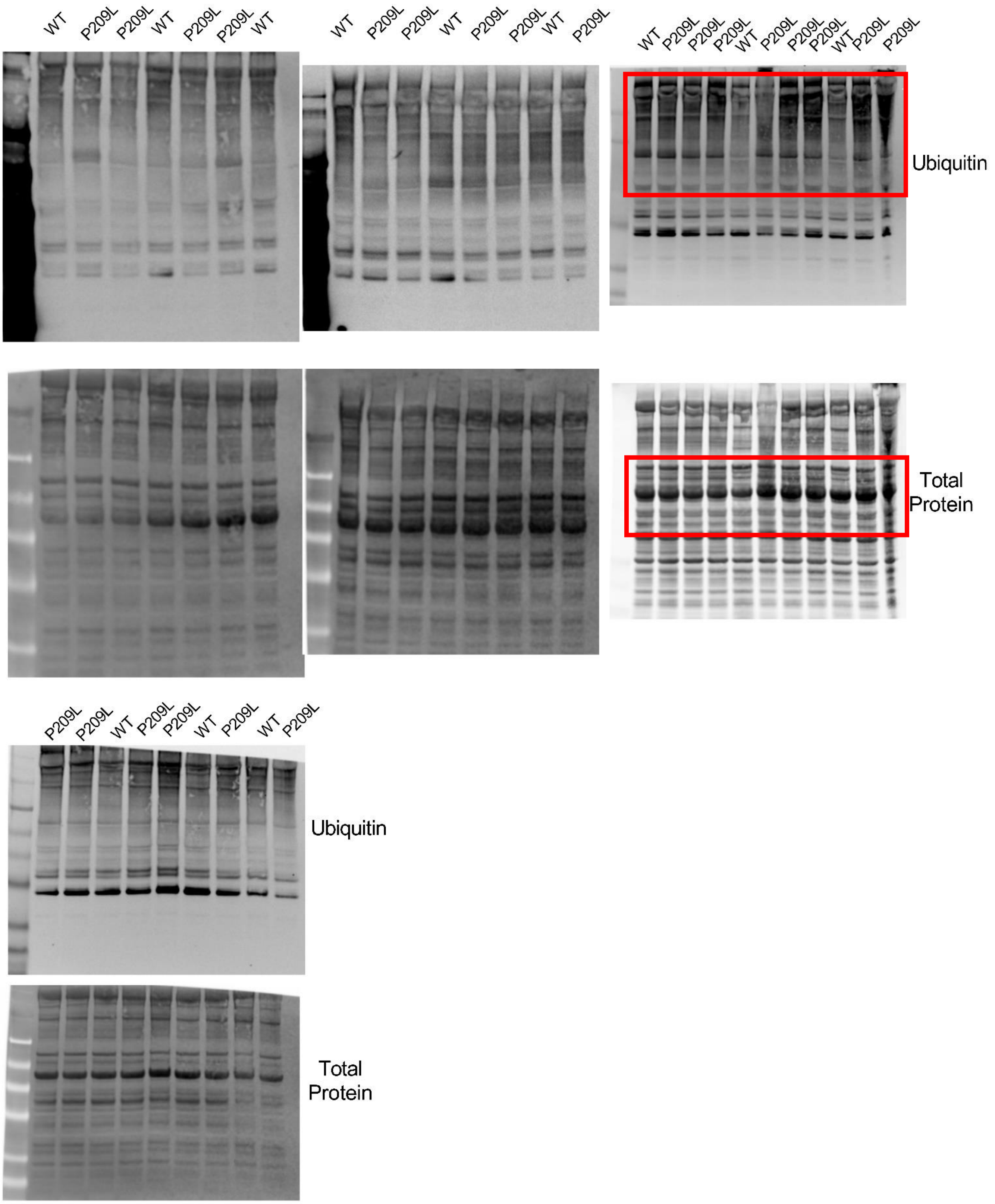

Uncropped western blots myofilament-specific ubiquitin in the WT and P209L mice pertaining to Supplementary Figure 4.

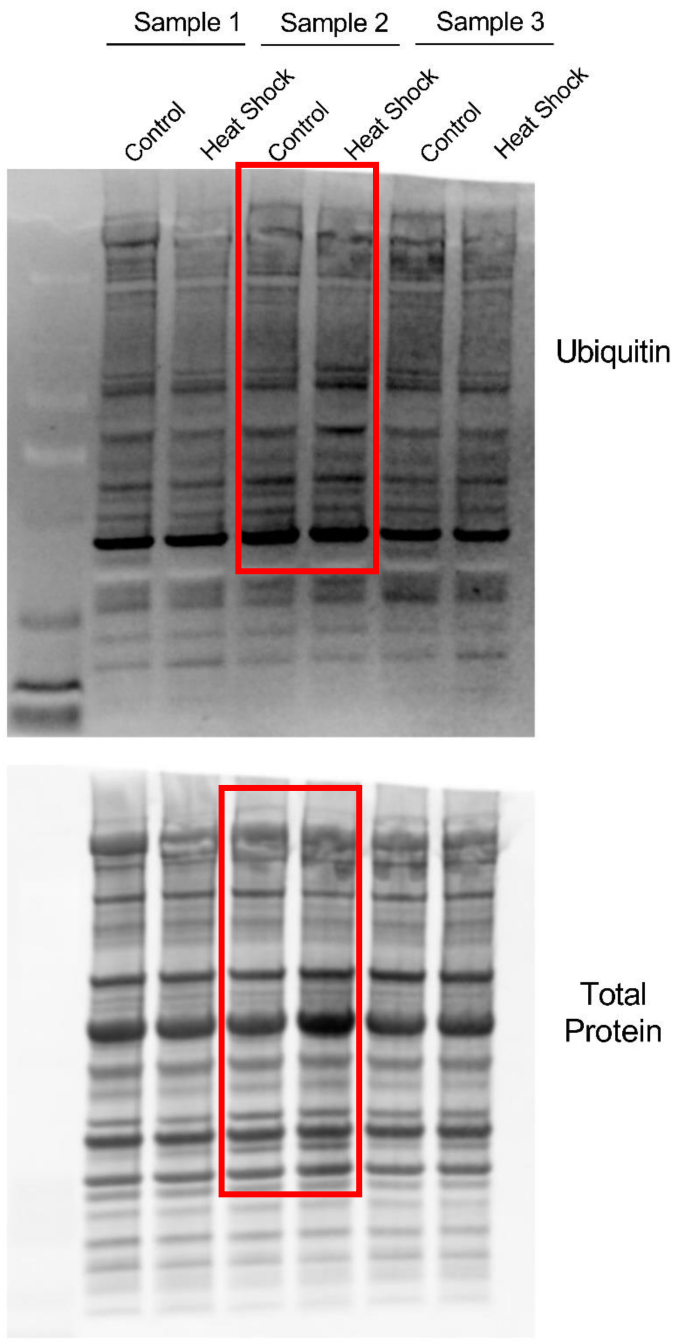

Uncropped western blot for myofilament-specific ubiquitin in the control and heat shock-treated skinned myocytes pertaining to Supplementary Figure 1.

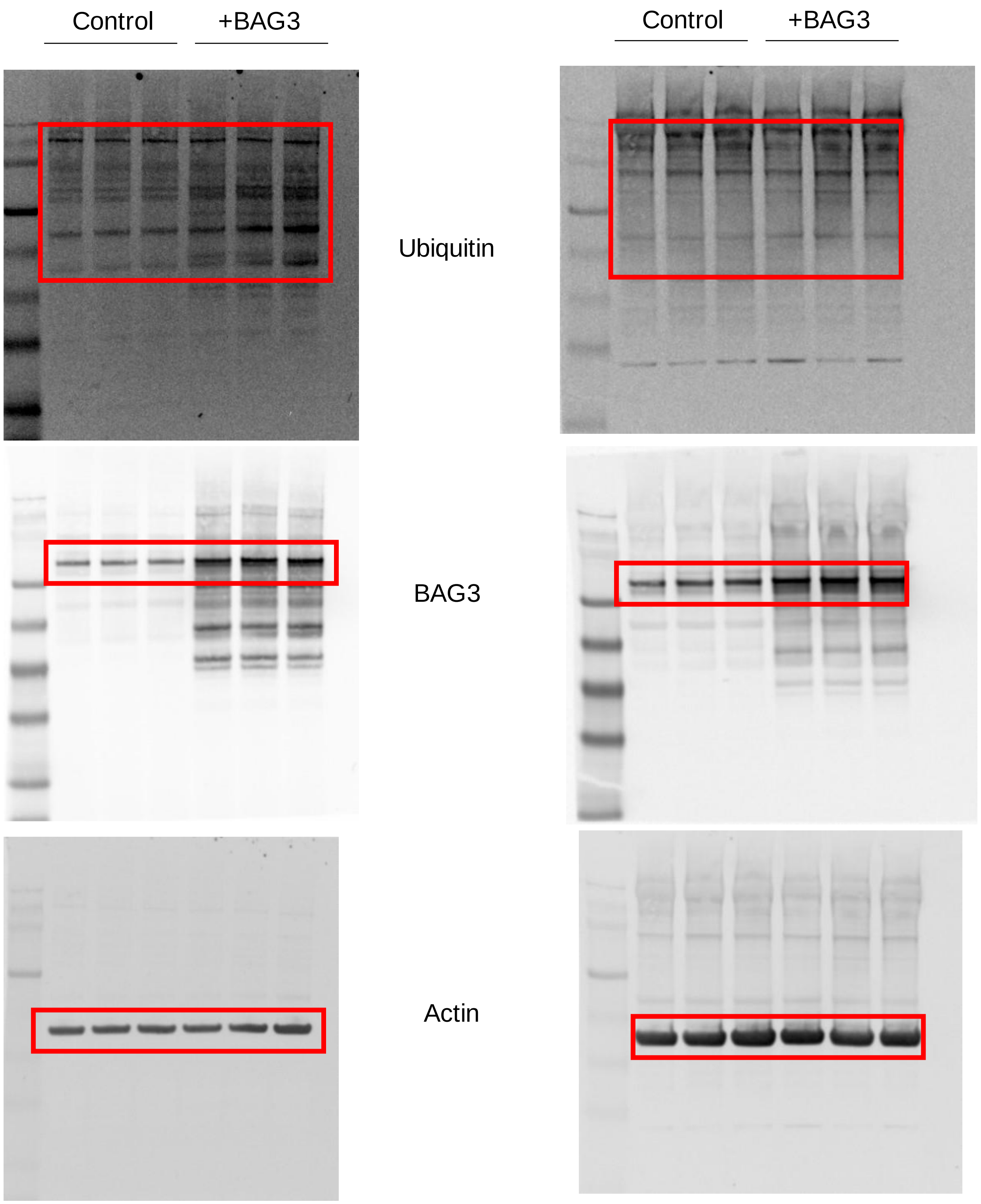

Uncropped western blot of the myofilament and released fraction from the client release experiment for ubiquitin, BAG3, and actin pertaining to Figure 6.
