# Supplementary material for: Cardiomyocyte contractile impairment in heart failure results from reduced BAG3-mediated sarcomeric protein turnover
Source: Nat Commun. 2021 May 19;12:2942. doi: 10.1038/s41467-021-23272-z (PMC8134551; doi:10.1038/s41467-021-23272-z)
Supplement: Supplementary file 3 — Descriptions of Additional Supplementary Files [file 41467_2021_23272_MOESM3_ESM.pdf]

Descriptions of Additional Supplementary Files

Supplementary Data 1
Description: CASA protein interactome identified by immunoprecipitation and LC-MS/MS.

Supplementary Data 2
Description: CASA myofilament client release identified by LC-MS/MS.

Supplementary Data 3
Description: Ubiquitinated peptides identified from ubiquitinenriched myofilament peptides by LCMS/MS.
