# Supplementary material for: Cardiomyocyte contractile impairment in heart failure results from reduced BAG3-mediated sarcomeric protein turnover
Source: Nat Commun. 2021 May 19;12:2942. doi: 10.1038/s41467-021-23272-z (PMC8134551; doi:10.1038/s41467-021-23272-z)
Supplement: Supplementary file 7 — Reporting Summary [file 41467_2021_23272_MOESM7_ESM.pdf]

nature research

Corresponding author(s): Kirk, Jonathan A

Last updated by author(s): Mar 17, 2021

Reporting Summary

Nature Research wishes to improve the reproducibility of the work that we publish. This form provides structure for consistency and transparency in reporting. For further information on Nature Research policies, see our Editorial Policies and the Editorial Policy Checklist.

Statistics

For all statistical analyses, confirm that the following items are present in the figure legend, table legend, main text, or Methods section.

n/a	Confirmed	
[ ]	[x]	The exact sample size (n) for each experimental group/condition, given as a discrete number and unit of measurement
[ ]	[x]	A statement on whether measurements were taken from distinct samples or whether the same sample was measured repeatedly
[ ]	[x]	The statistical test(s) used AND whether they are one- or two-sided<br>*Only common tests should be described solely by name; describe more complex techniques in the Methods section.*
[ ]	[x]	A description of all covariates tested
[ ]	[x]	A description of any assumptions or corrections, such as tests of normality and adjustment for multiple comparisons
[ ]	[x]	A full description of the statistical parameters including central tendency (e.g. means) or other basic estimates (e.g. regression coefficient) AND variation (e.g. standard deviation) or associated estimates of uncertainty (e.g. confidence intervals)
[ ]	[x]	For null hypothesis testing, the test statistic (e.g. F, t, r) with confidence intervals, effect sizes, degrees of freedom and P value noted<br>*Give P values as exact values whenever suitable.*
[x]	[ ]	For Bayesian analysis, information on the choice of priors and Markov chain Monte Carlo settings
[x]	[ ]	For hierarchical and complex designs, identification of the appropriate level for tests and full reporting of outcomes
[x]	[ ]	Estimates of effect sizes (e.g. Cohen's d, Pearson's r), indicating how they were calculated

Our web collection on statistics for biologists contains articles on many of the points above.

Software and code

Policy information about availability of computer code

Data collection	Azure c600 Imaging System, LI-COR Image Studio software, Zen Black Software (Zeiss), Fiji (ImageJ)
Data analysis	GraphPad Prism 8.0, LI-COR Image Studio Software

For manuscripts utilizing custom algorithms or software that are central to the research but not yet described in published literature, software must be made available to editors and reviewers. We strongly encourage code deposition in a community repository (e.g. GitHub). See the Nature Research guidelines for submitting code & software for further information.

Data

Policy information about availability of data

All manuscripts must include a data availability statement. This statement should provide the following information, where applicable:

- Accession codes, unique identifiers, or web links for publicly available datasets
- A list of figures that have associated raw data
- A description of any restrictions on data availability

The data in support of the findings of this study may be found within the manuscript and in the associated supplementary files. Mass spectrometry-based proteomics raw data are presented in Supplementary Data files 1-3 and were deposited in the MassIVE repository and linked to the ProteomeXchange Consortium with the dataset identifier PXD022414 [http://proteomecentral.proteomexchange.org/cgi/GetDataset?ID=PXD022414]. The raw data and uncropped western blot images pertaining to Figures 1a-c,e,i-n, 2b-d,f,h,j-l, 4c-g, 5b-d,f,h,j, 6d-m, 7a-c,e-h,j-k,m, and Supplemental Figures 1a-c,e, 2a-f, 3b-f, 4a-c,e,g, 5a-k, 6b-m are included in the accompanying Source Data File. Data associated with this study will be made available from the corresponding author upon reasonable request.

April 2020

Field-specific reporting

Please select the one below that is the best fit for your research. If you are not sure, read the appropriate sections before making your selection.

- [x] Life sciences
- [ ] Behavioural & social sciences
- [ ] Ecological, evolutionary & environmental sciences

For a reference copy of the document with all sections, see nature.com/documents/nr-reporting-summary-flat.pdf

Life sciences study design

All studies must disclose on these points even when the disclosure is negative.

Sample size	Where necessary, samples sizes were determined by power analysis. The metrics were: 20% change in the dependent variable, 80% power, and alpha of 0.05.
Data exclusions	No data were excluded in this study.
Replication	All in vitro experiments were replicated 3 or more times. For mouse and human tissue studies, experiments were replicated in 3 or more independent biological samples. The findings were successfully replicated in all attempts. Wherever possible we confirmed the study results using multiple models. Examples of this include the functional significance of BAG3 for the sarcomere, which we confirm by force-calcium experiments in four different models (3 mouse models, and human samples). We also use multiple methods to validate our findings of protein expression, association, and localization in the myofilament-specific fraction of cardiomyocytes. Most often, these include validation of findings achieved through one method using western blot, immunoprecipitation, immunofluorescence, or mass spectrometry.
Randomization	In experiments where treatments were used, such as for the mice that received LAD ligation and were subsequently treated with BAG3 gene therapy, the subjects/animals were randomly assigned to their treatment groups.
Blinding	Investigators were blinded during the data acquisition for the skinned cardiomyocyte force-calcium experiments. The blinding was removed during data analysis upon completion of the experiments. For molecular analyses, blinding was not deemed necessary as phenotypic differences between groups were readily apparent. In all cases, the analytical assessment was performed using unbiased approaches in order to limit experimenter bias.

Reporting for specific materials, systems and methods

We require information from authors about some types of materials, experimental systems and methods used in many studies. Here, indicate whether each material, system or method listed is relevant to your study. If you are not sure if a list item applies to your research, read the appropriate section before selecting a response.

Materials & experimental systems

n/a	Involved in the study	
[ ]	[x]	Antibodies
[x]	[ ]	Eukaryotic cell lines
[x]	[ ]	Palaeontology and archaeology
[ ]	[x]	Animals and other organisms
[ ]	[x]	Human research participants
[x]	[ ]	Clinical data
[x]	[ ]	Dual use research of concern

Methods

n/a	Involved in the study	
[x]	[ ]	ChIP-seq
[x]	[ ]	Flow cytometry
[x]	[ ]	MRI-based neuroimaging

Antibodies

Antibodies used	BAG3 (Proteintech, 10599-1AP), BAG3 (Santa Cruz Biotechnology, sc-136467), c-Myc (Cell Signaling Technology, 71D10), HspB8 (Proteintech, 15287-1AP), Hsp70 (Proteintech, 10995-1AP), Ubiquitin (Cytoskeleton Inc., AUB01), CHIP/STUB1 (Santa Cruz Biotechnology, sc-133083), CHIP (Proteintech, 55430-1-AP), P62/SQSTM1 (Proteintech, 18420-1AP), a-Actinin (Millipore Sigma, A7811), Oligomer A11 (Invitrogen, AHB0052), GAPDH (Cell Signaling Technology, 14C10), Sarcomeric a-actin (Millipore Sigma, A2172)
Validation	BAG3 rabbit polyclonal (Proteintech, 10599-1AP); validated with bottom-up mass spectrometry of immunoprecipitated protein and KO validated in this study; 64 citations on CiteAb https://www.citeab.com/antibodies/81478-10599-1-ap-bag3-antibody?des=772806a8e2dbb3a2<br>BAG3 mouse monoclonal (Santa Cruz Biotechnology, sc-136467); knockdown validated (Yu et al., PMID: 29484407); 4 citations on CiteAb https://www.citeab.com/antibodies/781720-sc-136467-bag-3-antibody-19?des=772806a8e2dbb3a2<br><br>c-Myc rabbit monoclonal (Cell Signaling Technology, 71D10); validated by identification of the myc-tagged BAG3 in our mouse model treated with AAV9-mycBAG3; 369 citations on CiteAb https://www.citeab.com/antibodies/123135-2278-myc-tag-71d10-rabbit-mab?des=104ccaac1de67b2c

HSPB8 rabbit polyclonal (Proteintech, 15287-1AP); validated with bottom-up mass spectrometry of immunoprecipitated protein; knockdown validated by Ramirez-Rodriguez et al. PMID: 23536091

HSP70 rabbit polyclonal (Proteintech, 10995-1AP); validated with bottom-up mass spectrometry of immunoprecipitated protein; 60 citations on CiteAb https://www.citeab.com/antibodies/976252-10995-1-ap-hsp70-antibody?des=25569944cbe1fb9a
Ubiquitin mouse monoclonal (Cytoskeleton Inc, AUB01 - clone P4D1); validated by identifiable increase in ubiquitin signal with proteasome inhibition; 2323 citations on CiteAb https://www.citeab.com/antibodies/828150-sc-8017-ubiquitin-antibody-p4d1?des=ef015a7067abdcfd

CHIP mouse monoclonal (Santa Cruz Biotechnology, sc-133083); knockdown validated by Yoo and Chung PMID: 29242192; 6 citations on CiteAb https://www.citeab.com/antibodies/811664-sc-133083-chip-antibody-c-10?des=5097c5c9862bc65b

CHIP rabbit polyclonal (Proteintech, 55430-1-AP); knockdown validated by Hu et al PMID: 33472166; 9 citations on CiteAb https://www.citeab.com/antibodies/981670-55430-1-ap-stub1-antibody?des=01b8361043cce8b4

P62/SQSTM1 rabbit polyclonal (Proteintech, 18420-1AP); validated by Dai et al PMID: 33416115 to increase in protein expression at appropriate molecular weight with autophagy flux inhibition; 562 citations on CiteAb https://www.citeab.com/antibodies/980164-18420-1-ap-p62-sqstm1-antibody?des=4278d560a1e10f53

a-Actinin mouse monoclonal (Millipore Sigma, A7811); validated by Z-disc localization; 851 citations on CiteAb https://www.citeab.com/antibodies/2281304-a7811-monoclonal-anti-alpha-actinin-sarcomeric-ant?des=81444bf884c3e46b
Oligomer A11 rabbit polyclonal (Invitrogen, AHB0052); validated by 70 citations on CiteAb https://www.citeab.com/antibodies/search?q=AHB0052

GAPDH rabbit monoclonal (Cell Signaling Technology, 14C10); validated by specific expression in cytosolic fraction; 3989 citations on CiteAb https://www.citeab.com/antibodies/122875-2118-gapdh-14c10-rabbit-mab?des=79bfdf6c9e98d061

a-Actin mouse monoclonal (Millipore Sigma, A2172); validated by myofilament specific expression; 61 citations on CiteAb https://www.citeab.com/antibodies/2304329-a2172-monoclonal-anti-actin-alpha-sarcomeric-antib?des=e9468885b5899241

Animals and other organisms

Policy information about studies involving animals; ARRIVE guidelines recommended for reporting animal research

Laboratory animals	Mouse HF Model: 8-week-old male and female C57Bl6/J; P209L Mouse Model: 8-month-old male and female C57Bl6/J; BAG3+/- and -/- Mice: 6-week-old male and female C57Bl6/J<br>Rats for primary ventricular myocyte experiments: One-day-old male and female Sprague-Dawley rats
Wild animals	No wild animals were used in this study.
Field-collected samples	No field-collected samples were used in this study.
Ethics oversight	All animal experiments were conducted in adherence to the US National Institutes of Health Guide for the Care and Use of Laboratory Animals. Experimental procedures were approved by the Temple University and Hines VA IACUC, which are AALAC accredited institutions. Mouse colonies were maintained in 12-hour light/dark cycles from 6 AM to 6 PM at 65-75 °F/40-50% humidity, and had unrestricted access to food and water. Neonatal rat ventricular myocyte studies were approved by the Loyola University Chicago Health Science Division IACUC, which is an AALAC accredited institution.

Note that full information on the approval of the study protocol must also be provided in the manuscript.

Human research participants

Policy information about studies involving human research participants

Population characteristics	All human samples used were obtained from the Loyola University Chicago Cardiovascular Research Institute and Cleveland Clinic tissue biobanks. The tissue was de-identified at the time of banking and assigned a sample number. All relevant patient characteristics (i.e. age, sex, ethnicity, left ventricular ejection fraction) are included within the manuscript in Table 1.
Recruitment	Not applicable. These samples came from tissue biobanks and were not recruited specifically for this study.
Ethics oversight	Informed consent was obtained prior to tissue collection, which was obtained with permission from the Institutional Review Boards at Loyola University Chicago Health Science Division and the Cleveland Clinic.

Note that full information on the approval of the study protocol must also be provided in the manuscript.
